# When social identities intersect: understanding inequities in growth outcomes by religion- caste and religion-tribe as intersecting strata of social hierarchy for Muslim and Hindu children in India

**DOI:** 10.1186/s12939-023-01917-3

**Published:** 2023-06-14

**Authors:** Pritha Chatterjee, Jarvis Chen, Aisha Yousafzai, Ichiro Kawachi, S. V. Subramanian

**Affiliations:** 1 Department of Social and Behavioral Sciences, Harvard T.H. Chan School of Public Health, 677 Huntington Avenue, Boston, MA 02115 USA; 2 Department of Global Health and Population, Harvard T.H. Chan School of Public Health, 677 Huntington Avenue, Boston, MA 02115 USA; 3 Harvard Center for Population and Development Studies, Cambridge, MA 02138; and, Department of Social and Behavioral Sciences, Harvard T.H. Chan School of Public Health, MA 02115, Boston, USA

**Keywords:** Caste, Religion, Intersectionality, Child health disparity, Health inequity, India, South-Asia

## Abstract

**Background:**

Minority social status determined by religion, caste and tribal group affiliations, are usually treated as independent dimensions of inequities in India. This masks relative privileges and disadvantages at the intersections of religion-caste and religion-tribal group affiliations, and their associations with population health disparities.

**Methods:**

Our analysis was motivated by applications of the intersectionality framework in public health, which underlines how different systems of social stratification mutually inform relative access to material resources and social privilege, that are associated with distributions of population health. Based on this framework and using nationally representative National Family Health Surveys of 1992–93, 1998–99, 2005–06, 2015–16 and 2019–21, we estimated joint disparities by religion-caste and religion-tribe, for prevalence of stunting, underweight and wasting in children between 0–5 years of age. As indicators of long- and short-term growth interruptions, these are key population health indicators capturing developmental potential of children. Our sample included Hindu and Muslim children of <  = 5 years, who belonged to Other (forward) castes (the most privileged social group), Other Backward Classes (OBCs), Schedule Castes (SCs) and Schedule Tribe (STs). Hindu-Other (forward) caste, as the strata with the dual advantages of religion and social group was specified as the reference category. We specified Log Poisson models to estimate multiplicative interactions of religion- caste and religion-tribe identities on risk ratio scales. We specified variables that may be associated with caste, tribe, or religion, as dimensions of social hierarchy, and/or with child growth as covariates, including fixed effects for states, survey years, child’s age, sex, household urbanicity, wealth, maternal education, mother’s height, and weight. We assessed patterns in growth outcomes by intersectional religion-caste and religion-tribe subgroups nationally, assessed their trends over the last 30 years, and across states.

**Findings:**

The sample comprised 6,594, 4,824, 8,595, 40,950 and 3,352 Muslim children, and 37,231, 24,551, 35,499, 1,87,573 and 171,055 Hindu children over NFHS 1, 2, 3, 4, and 5, respectively. As one example anthropometric outcome, predicted prevalence of stunting among different subgroups were as follows- Hindu Other: 34.7% (95%CI: 33.8, 35.7), Muslim Other: 39.2% (95% CI: 38, 40.5), Hindu OBC: 38.2 (95%CI: 37.1, 39.3), Muslim OBC: 39.6% (95%CI: 38.3, 41), Hindu SCs: 39.5% (95%CI: 38.2, 40.8), Muslims identifying as SCs: 38.5% (95%CI: 35.1, 42.3), Hindu STs: 40.6% (95% CI: 39.4, 41.9), Muslim STs: 39.7% (95%CI: 37.2, 42.4). Over the last three decades, Muslims always had higher prevalence of stunting than Hindus across caste groups. But this difference doubled for the most advantaged castes (Others) and reduced for OBCs (less privileged caste group). For SCs, who are the most disadvantaged caste group, the Muslim disadvantage reversed to an advantage. Among tribes (STs), Muslims always had an advantage, which reduced over time. Similar directions and effect sizes were estimated for prevalence of underweight. For prevalence of wasting, effect sizes were in the same range, but not statistically significant for two minority castes-OBCs and SCs.

**Interpretation:**

Hindu children had the highest advantages over Muslim children when they belonged to the most privileged castes. Muslim forward caste children were also disadvantaged compared to Hindu children from deprived castes (Hindu OBCs and Hindu SCs), in the case of stunting. Thus, disadvantages from a socially underprivileged religious identity, seemed to override relative social advantages of forward caste identity for Muslim children. Disadvantages born of caste identity seemed to take precedence over the social advantages of Hindu religious identity, for Hindu children of deprived castes and tribes. The doubly marginalized Muslim children from deprived castes were always behind their Hindu counter parts, although their differentials were less than that of Muslim-Hindu children of forward castes. For tribal children, Muslim identity seemed to play a protective role. Our findings indicate monitoring child development outcomes by subgroups capturing intersectional social experiences of relative privilege and access from intersecting religion and social group identities, could inform policies to target health disparities.

**Supplementary Information:**

The online version contains supplementary material available at 10.1186/s12939-023-01917-3.

## Introduction

### Overview

Health disparities determined by social hierarchies are usually assessed along one of three axes in India: religion, caste and tribe. In policy and research, scant attention is accorded to health inequities associated with intersectional lived experiences of religion and caste, and religion and tribe, as simultaneous measures of social position [1]. Treating these axes independently has the implicit theoretical assumption that patterns of social advantage and disadvantage determined by each of these social structures are unrelated in their influence on health disparities [2]. However, lived social experiences of people facing accrued disadvantages determined by one or more of these social hierarchies are likely multiple and simultaneous [3–5].

India is home to 79.8% Hindus, 14.2% Muslims, 2.3% Christians, 1.7% Sikhs, 0.7% Buddhists [6]. That descent based social stratification among Muslims, Christians, Sikhs and Buddhists is replete with features of the Hindu caste system, is discussed extensively in the social science and humanities literature [4, 5, 7]. We focus this analysis on Hindus, as the majority religious group, and Muslims, as the largest religious minority in India. However, our approach to study health inequities by social strata formed by intersections of religion and caste, and religion and tribes could also be extended to Christians.

Broadly, Muslims in India hail from three hierarchical social groups- the Ashrafs, believed to be of Persian or Arab heritage, who were traditionally landowners or wealthy businessmen; the Ajlafs, largely converts of middle or higher Hindu castes, historically engaged in farming, trading etc. and the Arzals, who converted from lower or “untouchable” castes outside of the traditional Hindu *varna* system [5]. Thus, due to the sociopolitical context of the region, social stratification along caste lines is integrated with the Muslim identity, in India, and more broadly, South Asia. However, epidemiological analysis from the region tends to treat all faiths barring Hinduism as singular, monolithic identities, distinct from caste [8]. Similarly, tribes across the country adhere to different faiths including Islam and Christianity [9]. Muslim tribes primarily live in Jammu and Kashmir; and in Maharashtra and Lakshadweep [9, 10]. However, the scant epidemiology focused on tribal health disparities usually does not consider the intersections of tribal social group in conjunction with religious identity as an interlocked social category [8].

While these intersectional social disparities can be studied in their influence on any population health patterns, we examine them in the case of three outcomes associated with growth and development of children under 5 years: stunting, wasting and underweight [11]. Jointly, they allow us to examine how privileges associated with these intersecting systems of social stratification are associated with inequities in current and future developmental potential of children in India.

### Overview of social groups in India: caste, tribes and their intersections with religion as dimensions of social identity

In this section, we provide an overview of caste and tribe, the two axes we are calling “social groups” in this analysis. In the case of caste, we discuss two typologies of backward communities. For tribes, we provide details of deprived communities identified as protected tribes. We also present details of how caste and tribe intersect with the two religious groups we are focusing on-Hindus and Muslims.

First, “backward” castes, historically deemed “untouchable” and prohibited from any proximity to superior castes were classified as Schedule Castes and accorded constitutional safeguards in 1950 [12]. Barring minor changes, this ‘schedule’ or list of communities has largely remained unchanged in India. Despite the existence of constitutionally mandated legal protections in access to education and employment since India’s independence, communities identified as Schedule Castes continue to be deprived in economic and educational indicators [13]. While this schedule, or list of castes historically included communities from both Hindu and Muslim religions [7, 14], in an amendment from 1950, it was restricted to Hindus, with the rationale that other religions did not suffer the social ills of the Hindu caste system [14]. However, this decision contradicted historical censuses conducted under colonially administered India of 1901, 1911,1921 and 1931, where data on castes among Muslims were routinely collected and included in identification of backward caste status [14]. In contemporary India, while Muslims hailing from these backward castes cannot legally be recognized as Schedule Castes and are therefore not guaranteed affirmative action policies in education and employment available to their Hindu counterparts, many government commissioned reports have described the socioeconomic backwardness of Muslims who identify as Schedule Castes [15, 16]. A 2007 National Minority Commission report noted these Muslim communities are “socially known and treated as distinct groups”, and that often, their caste status takes precedence over their religion, in the patterning of their economic, health and educational outcomes [15, 17].

Other Backward Classes (OBC) are a second category of deprived communities associated with caste identity. The “Other” here, refers to about 2500 “socially and economically backward” communities that were historically not “untouchable” castes, but deprived in eleven socioeconomic criteria including access to improved housing and drinking water, family assets, age at marriage, female work participation, school drop-out rate, among others [18]. Importantly, communities from all faiths can be legally recognized as Other Backward Classes, and are guaranteed some affirmative action policies. However, these constitutional protections are not as exhaustive as in the case of Schedule Castes [14]. The list of communities recognized under the Other Backward Classes category also varies by states, with some communities granted this deprived community status in some states, but not others [14]. Furthermore, in the absence of Schedule Caste status, this social category is the only route to affirmative action for Muslims of deprived castes [14]. Thus, Muslim Other Backward Classes may comprise of erstwhile Hindu Schedule Castes who have been legally granted the Other Backward Class status in some states. Some Muslim communities recognized under the Other Backward Class category are also communities who are equivalent to Hindu Other Backward Classes [14].

The residual “other” castes comprise of communities that are “forward castes”, or “upper castes” in India. Here, “forward” and “upper” are not legally recognized terms, but are routinely used in scholarship on caste, political discourse, and popular parlance in India. While Hindu other (forward) castes have historically been associated with the highest socioeconomic status and intergenerational mobility, as well as most social privilege and power in India [19], Muslim forward castes also enjoy the most advantaged social position in the religion [20]. However, their caste affiliation notwithstanding, Muslims as a community lag Hindus in education, wealth and other socioeconomic and health related indicators [21, 22]. Since religion is not a constitutionally recognized criteria of affirmative action policies in India, Muslims as a whole, have not benefited from such social safeguards, despite political and legal debates around this [14]. However, as stated above, Muslims who hail from castes who are recognized as Other Backward Classes are granted some of these safeguards in limited states, due to their Other Backward Class status [14].

Finally, around 740 tribes who were the earliest settlers in the Indian subcontinent were recognized as tribes during the British rule. These communities were re-classified as Schedule Tribes in independent India in 1950 [10]*.* Their characteristics, as defined under the Indian constitution, include “primitive traits, geographical isolation, distinct culture, shyness of contact, and economic backwardness” [9]. Schedule Tribe status can be accorded to tribes from all religions, such that nearly 80 million tribes in India identify as Hindus or Buddhists, abouts 1.2 million as Muslims, and nearly 8 million as Christians [9, 10]. While tribes recognized as Schedule Tribes have been granted affirmative action policies since India’s independence, and many tribal welfare policies have been explicitly targeted to improve their educational outcomes, these communities continue to be deprived in socioeconomically, and in health and educational metrics [9]. They also largely continue to live in remote forest regions, with poor access to health and other infrastructure [9].

For the rest of this paper, we will be using the following acronyms that are commonly used in India to discuss these caste and tribal social groups. We will refer to Schedule Castes as SCs, Other Backward Classes as OBCs, and Schedule Tribes as STs. We will refer to the privileged social group comprising of advantaged castes and/or non-tribes as Other (Forward) castes. These social groups are summarized in Table S1.

### Conceptualizing religion- caste and religion-tribe as simultaneous and interacting dimensions of social inequities

In India and South Asia, caste, tribe and religion have historically interacted and mutually reinforced deep rooted societal hierarchies that determine access to wealth, education, power and intergenerational mobility for generations [23]. These interlocking social identities are jointly associated with multiple structural exposures which inform health disparities, including access to material resources and education, occupation, nutritional support, exposure to community violence, and migration patterns [24].

Intersectionality is a theoretical framework that posits that multiple social categories intersect and synergistically influence structures of privilege and oppression [2]. Rooted in Black feminist scholarship, it underscores the embodied experiences of multiple simultaneous social positions, which concurrently determine access to power and resources in any society [25]. Bowleg provides a helpful framework in the application of intersectionality to public health, in positing that different socially constructed dimensions of social identities “constitute each other” [2]. Thus, treating them as independent obfuscates health disparities associated with experiences at intersections of these social hierarchies [2, 26]. These tenets of intersectionality are inherent to social epidemiology’s fundamental exposition to measure patterns of social context, to understand distributions of population health outcomes [27].

Thus, based on the intersectionality framework, we hypothesized that deprived caste or tribal identity may not accord similar relative disadvantages in health outcomes to Hindu and Muslim children and vice versa, due to the simultaneous lived social experiences of backward caste or tribal identity with minority religious identity. In this exposition, we examined caste, tribe and religion as dimensions of social identity, that collectively determine social hierarchies in Indian society [19]. In exploring this hypothesis, we contribute to the intersectionality literature in three ways. First, we apply the intersectionality framework to a non-western context [28]. Second, we investigate minority religious identity as a dimension of social identity in the intersectionality framework, in response to recent calls about its potentially important role in understanding disparities multireligious societies, and for improving understanding of intersectional disparities patterned by ‘traditional’ social strata like income, education, gender, among others [26, 27, 29]. Third, while most public health applications of the intersectionality framework are focused on adult or life course epidemiological outcomes, we apply it in the context of child development [30]. In this, we draw from the Ecological Systems Theory of child development, which posits that children are embedded in multiple interacting social and physical environments, and contextually relevant diverse systems of privilege and deprivation determine their developmental context [31–33]. Thus, we use the intersectionality framework to quantitatively measure the social context of development for Hindu and Muslim children of different social groups in India, in their associations with growth outcomes.

A growing literature largely focused on the study of structural inequities associated with racial discrimination, has dwelled on methodological considerations for the quantitative study of intersectional health inequities in population health research [26, 34, 35]. We followed Jackson’s three-way decomposition approach, which quantitatively decomposes disparities faced by jointly marginalized groups, into disparities associated with each constituent marginalization, and disparities associated with their intersections [36]. It has a key assumption that disparities associated with social hierarchies are a reflection of discrimination and social deprivations associated with these social identities [35]. For example, in its application in the United States, health disparities among racial and ethnic minority, queer women of color have been decomposed into disparities associated with racial discrimination, homophobia, and the intersections of racial discrimination and homophobia [36, 37]. This method is especially suited to the estimation of intersectional disparities associated with two dimensions of social stratification [35, 36]. Thus, it was well suited to our purposes of studying how intersectional social privileges associated with simultaneous religion-social group affiliations were associated with child health disparities in India.

While our primary research question is focused on social positions jointly informed by intersections of religion and caste, and religion and tribe, in keeping with the historical roots of the theoretical framework of intersectionality, we also explore how these social strata may interact with other systems of social stratification, in informing patterns of health disparities. For example, caste as a social construct is inherently patriarchal, with deep rooted gendered problems like a son preference leading to depleting sex ratios among Hindus in parts of the country, as well as in its strong influence in gender norms resulting in poor literacy and access to health for women from deprived castes [38, 39]. Minority caste women also face larger barriers in access to health and well-being and poor maternal health outcomes. At the same time, Muslim women lag Hindu women in literacy, education and access to health [40]. Historically, women are also subject to violence in the name of caste and religion when any type of intercommunity strife breaks out [41]. Patterns of wealth, land ownership and literacy are associated with both caste and religion, such that deprived castes and Muslim minorities are poorer, own less assets and have lower literacy and poor education levels [42]. Thus, we also sought to study how strata formed by each of household wealth, education, child’s gender and age, intersect with social strata formed by religion and social group identities, in their association with patterns of child growth.

### Child growth outcomes as indicators of children’s developmental potential

We focused on three child growth outcomes as indicators of current and future developmental potential of children. While stunting is an indicator of longer-term disruptions in growth and nutrition, informed by accumulated adversities, wasting is reflective of short term interruptions in children’s nutrition and development [43]. Underweight captures both short- and long-term disruptions. In India, despite many targeted of national nutrition sensitive and nutrition specific interventions, the prevalence of these outcomes in children under 5 years of age, continues to be high, with 36% children stunted, 19% wasted and 32% underweight [44]. Thus, together, these outcomes allow us to explore how intersectional patterns of caste and religion based social hierarchies are associated with disparities for Hindu and Muslim children in India.

Finally, as outlined above, affirmative action policies for the upliftment of deprived castes and tribes have been in place since India’s independence in 1947 [45]. Parallel to this, debates over the social and economic backwardness of Muslims have continued, while Muslim deprived caste communities have also organized as a unique political group demanding representation and affirmative action policies granted to fellow deprived castes communities from other religions [14, 22]. Given this historical precedence, we also sought to study how our hypothesized patterns of intersectional disparities in children’s stunting, wasting and underweight have varied over the last 30 years, and across states.

## Methods

### Data

We used data on sampled Hindu and Muslim children from National Family Health Surveys (NFHS) of 1992–93 (NFHS 1), 1998–99 (NFHS 2), 2005–06 (NFHS 3), 2015–16 (NFHS 4) and 2019–21 (NFHS 5) which measured height, weight and of children <  = 5 years [44]. The survey was representative at state level until NFHS 3, and at district level in NFHS 4 and 5. The OBC category was not officially recognized during data collection for NFHS 1 [46]. NFHS 3 only collected anthropometric data for children < 3 years of age. All surveys were based on stratified two stage random sampling designs, with census enumeration blocks and villages as the primary sampling units in urban and rural areas respectively, based on the latest census as the sampling frames [44].

### Exposure

The exposure of interest was the interaction of religious and caste or tribal groups, resulting in the following religion-caste strata: Hindu-Other (forward) caste, Muslim-Other (forward) caste, Hindu- Schedule Castes (SC), Muslims identifying as SC, Hindu Other Backward Class (OBC) and Muslim OBC, as well as two religion-tribe strata: Hindu Schedule Tribes (STs) and Muslim STs. While these intersectional social strata comprised our primary exposure, we also assessed their interactions with four other axes of social stratification that are associated with religion-caste and religion-tribe as social identities, and our outcomes of interest, and could thus potentially inform intersectional patterns of child health disparities. These included household wealth, maternal education, child’s gender, and child’s age.

### Outcomes

We assessed anthropometric outcomes defined as per WHO child reference standards of z-scores of <  = -2 for height-for-age (moderate stunting), weight-for-age (moderate underweight) and weight-for-height (moderate wasting) [47]. Absolute values of above 6 in these values were specified as missing. Stunting or low HAZ reflects cumulative effects of chronic undernutrition accrued since conception that may reflect the child’s long term growth potential [48]. Wasting or low WHZ indicates acute undernutrition from insufficient food intake or a high incidence of infectious diseases [48]. Underweight or low WAZ can be a result of both wasting and/or stunting [11].

### Covariates

We included as covariates, variables that have been associated with the exposure or outcomes of interest, or both, and those that met the theoretical conceptualization of caste, tribe and religion as axes of social inequality, determining access to power and resources [49]. Several child, maternal, household, environmental, and socioeconomic factors have been identified as predictors of anthropometric outcomes [11, 50, 51]. Of these, household wealth, maternal stature and maternal education have the strongest associations [50], such that children from poorer households, who are born to less educated mothers, or mothers with lower stature have higher prevalence of all three outcomes, but especially stunting [11, 51]. Maternal stature has been identified as a strong predictor of advantageous social and material position which may be transmitted intergenerationally [11]. Beyond individual and household level variables, place, particularly state of residence, has also been identified as a predictor due to varying spending on health and child development and different socioeconomic and health indicators across states [50, 52]. Additionally, within state heterogeneities by district and degree of urbanicity has also been associated with child growth outcomes [52]. At the same time, caste, tribe and religious identity as indicators of social stratification have historically determined access to wealth, education, power and intergenerational income and educational mobility [53, 54]. Thus, the covariates spread across three ecological levels- state (urbanicity, state and district fixed effects), household (mother’s height (for stunting and wasting), mother’s weight (for underweight and wasting), and child (child’s age and sex). The household wealth index was constructed by Principal Component Analysis of measures of living standards and asset ownership [55]. Maternal education was classified as no schooling, primary, secondary, higher secondary schooling, and college education or above. Mother’s height was categorized as < 145, 145–149.9, 150–154.9, 155–159.9, and 160 + cm. We also included each NFHS survey wave as a fixed effect to control for all state invariant factors that may vary over time.

### Statistical analysis

We followed Jackson’s three-way decomposition approach, which decomposes joint disparities or disparities faced by doubly marginalized groups, into disparities associated with each component disparity alone [36]. In our case, this estimates the joint disparity of social marginalization associated with deprived caste or tribal identity, and minority religious identity, synergistically. Disparities associated caste or tribe and Muslim identity independently are termed the “referent disparities” [36]. In statistical terms, these referent disparities are the “main effects” associated with each dimension of social stratification [37]. The estimated interaction effect of both social identities is known as the “excess intersectional disparity” associated by the intersectional experiences of deprivation associated with the joint consideration of these social identities [35].

Based on this decomposition approach, and following Knol and VanderWeele’s guidelines for epidemiological analysis of interaction effects, we estimated intersectional interaction effects associated with minority religion (Muslim) and socially disadvantaged caste and tribal identities, on both multiplicative and additive scales [56]. Since the prevalence of each anthropometric outcome within strata of covariates was above 10%, which was above the generally acceptable threshold of a rare outcome [57], and Log Binomial models failed to converge, we specified Log Poisson models [57] to estimate multiplicative interactions on risk ratio scales. Additive interactions were estimated by the relative excess risk due to interaction (RERI), which assesses the total effect due to interaction [58]. A RERI = 0 means no interaction, a RERI > 0 means a positive interaction or more than additivity; and a RERI < 0 means a negative interaction [58]. We calculated 95% Confidence Intervals (CIs) using the delta method [56] with cluster robust standard errors. All analyses were weighted by NFHS provided survey weights [44].

As per Knol and VanderWeele’s recommendations in estimating interaction effects, and based on Jackson and VanderWeele’s approach in the decomposition of intersectional inequities, we specified the stratum with the lowest risks of each outcome, Hindu-Other (Forward) castes, as the reference social strata [36, 56, 59]. This social stratum is also historically the most socioeconomically privileged, associated with the highest intergenerational economic mobility, and identified in most positions of occupational privilege [40]. We present Risk Ratios (RRs) with CIs for the association of social groups (caste or tribes) and outcomes within strata of religion, and religion and outcomes within strata of social groups (caste or tribes), with raw data on the number of subjects with and without the outcome in each cell as recommended under Strengthening the reporting of observational studies in epidemiology (STROBE) guidelines for presentation of interaction estimates (Table S3) [56].

To assess average within state differences for the interaction of religion and social group (caste or tribe) across all states, and control for time invariant state level factors, we included fixed effects for states in our national analysis. In estimating state trends, to allow for different strengths of associations for the interaction of religion and caste/tribal identity across states, we estimated two level random effects models, with random intercepts for states and random slopes for the interaction of caste and religion.

Finally, to estimate how other axes of social stratification inform intersectional patterns of child growth by religion-caste and religion-tribe as social identities, we estimated three-way interactions of child’s religion-caste or religion-tribe strata, with each of the following variables: child’s household wealth, maternal education, child’s gender, and child’s age. We also calculated 95% CIs with cluster robust standard errors, and weighted our estimates by NFHS provided survey weights [44]. All analysis was performed in RStudio version 4.1.2.

### Sensitivity analysis

We estimated unadjusted interactions for religion and caste on child anthropometry, to assess how far our covariates explained the religion-social group associated disparities (Table S2). This was because some covariates like household wealth and maternal education could be mediators in how intersectional social group experiences influence health outcomes (Table S2). Since the composition of OBCs has changed over time, we also restricted the sample to NFHS 4 and 5, when the distribution of sampled children across castes/tribes were largely consistent (Figure S1). Finally, since some deprived castes may be recognized as OBCs in some states, but others we also assessed how our national intersectional patterns for OBCs of both religions varied by state patterns (Fig. 3).

## Results

### Descriptive statistics

The sample comprised 6,594, 4,824, 8,595, 40,950 and 3,352 Muslim children, and 37,231, 24,551, 35,499, 1,87,573 and 171,055 Hindu children between NFHS 1–5 respectively (Table 1). Across survey waves, the smallest sampled subgroups were Muslim identifying as SCs and Muslim STs (Table 1). In each wave, more Hindu Other castes hailed from urban areas, while Muslims had higher urbanicity for all other castes/tribes (Table 1). More Muslim children had mothers without any education across castes/tribes, barring STs. In NFHS 5, 24.6% Muslims against 9.2% Hindu Other castes, 33.1% Muslim against 19.5% Hindu OBC, 40.1% Muslim SCs against 25.5% Hindu SC children, and 29.4 Muslim ST children compared to 35.2% Hindu ST children, had mothers with no education (Table 1). A higher percentage of Muslims belonged to poorest wealth quintiles, with the highest Hindu-Muslim gaps in wealth between Other (forward) castes and STs (Table 1). However, for Other (forward) castes, more Hindus belonged to the richest wealth quintile, but for OBCs and STs, more Muslims hailed from the richest households (Table 1).

**Table 1:** Distribution of socioeconomic and demographic characteristics for religion-social group subgroups for Hindu-Muslim children by waves of National Family Health Surveys in India

	**NFHS 1**								
	**Hindu (** ***n*** ** = 37,231)**				**Muslim (** ***n*** ** = 6594)**				
**Variable**	**Other**	**Other Backward Class**	**Scheduled Caste**	**Scheduled Tribe**	**Other**	**Other Backward Class**		**Scheduled Caste**	**Scheduled Tribe**
**N**	**27,467**	**NA**	**5903**	**3861**	**6351**	**NA**		**201**	**42**
A) NFHS 1^a^									
** Age of child (mean (SD))**	1.46 (1.12)	NA	1.45 (1.14)	1.49 (1.14)	1.47 (1.12)	NA		1.48 (1.17)	1.44 (1.08)
* Missing*	*2048 (7.5%)*	*NA*	*571 (9.7%)*	*365 (9.5%)*	*448 (7.1%)*	*NA*		*20 (10.0%)*	*3 (7.1%)*
**Sex of Child = male (%)**									
Male	14,123 (51.4%)	NA	3061 (51.9%)	1986 (51.4%)	3214 (50.6%)	NA		108 (53.7%)	25 (59.5%)
Female	13,344 (48.6%)	NA	2842 (48.1%)	1875 (48.6%)	3137 (49.4%)	NA		93 (46.3%)	17 (40.5%)
* Missing*	*0 (0.0%)*	*NA*	*0 (0.0%)*	*0 (0.0%)*	*0 (0.0%)*	*NA*		*0 (0.0%)*	*0 (0.0%)*
**Mother’s Education (%)**									
No education	15,039 (54.8%)	NA	4614 (78.2%)	3348 (86.7%)	4132 (65.1%)	NA		168 (83.6%)	37 (88.1%)
Primary	4491 (16.4%)	NA	687 (11.6%)	274 (7.1%)	1120 (17.6%)	NA		17 (8.5%)	2 (4.8%)
Secondary	6499 (23.7%)	NA	554 (9.4%)	216 (5.6%)	993 (15.6%)	NA		16 (8.0%)	3 (7.1%)
Higher	1354 (4.9%)	NA	33 (0.6%)	11 (0.3%)	67 (1.1%)	NA		0 (0.0%)	0 (0.0%)
* Missing*	*84 (0.3%)*	*NA*	*15 (0.3%)*	*12 (0.3%)*	*39 (0.6%)*	*NA*		*0 (0.0%)*	*0 (0.0%)*
**Wealth quintile (%)**									
Poorest	4718 (17.2%)	NA	1352 (22.9%)	1647 (42.7%)	1008 (15.9%)	NA		33 (16.4%)	6 (14.3%)
Poorer	4871 (17.7%)	NA	1369 (23.2%)	981 (25.4%)	1342 (21.1%)	NA		43 (21.4%)	11 (26.2%)
Middle	5494 (20.0%)	NA	1306 (22.1%)	756 (19.6%)	1244 (19.6%)	NA		44 (21.9%)	11 (26.2%)
Richer	6270 (22.8%)	NA	1336 (22.6%)	337 (8.7%)	1499 (23.6%)	NA		40 (19.9%)	11 (26.2%)
Richest	6114 (22.3%)	NA	540 (9.1%)	140 (3.6%)	1258 (19.8%)	NA		41 (20.4%)	3 (7.1%)
* Missing*	*0 (0.0%)*	*NA*	*0 (0.0%)*	*0 (0.0%)*	*0 (0.0%)*	*NA*		*0 (0.0%)*	*0 (0.0%)*
**Mother's height (mean (SD))**	NA	NA	NA	NA	NA	NA		NA	NA
* Missing*	*NA*	*NA*	*NA*	*NA*	*NA*	*NA*		*NA*	*NA*
**Urban/ Rural (%)**									
Urban	7846 (28.6%)	NA	1226 (20.8%)	331 (8.6%)	2285 (36.0%)	NA		48 (23.9%)	4 (9.5%)
Rural	19,621 (71.4%)	NA	4677 (79.2%)	3530 (91.4%)	4066 (64.0%)	NA		153 (76.1%)	38 (90.5%)
* Missing*	*0 (0.0%)*	*NA*	*0 (0.0%)*	*0 (0.0%)*	*0 (0.0%)*	*NA*		*0 (0.0%)*	*0 (0.0%)*
	**NFHS 2**								
	**Hindu (** ***n*** ** = 24,551)**				**Muslim (** ***n*** ** = 4824)**				
**Variable**	**Other**	**Other Backward Class**	**Scheduled Caste**	**Scheduled Tribe**	**Other**	**Other Backward Class**		**Scheduled Caste**	**Scheduled Tribe**
**n**	**8304**	**7987**	**5562**	**2698**	**3614**	**1041**		**119**	**50**
B) NFHS 2									
** Age of child (mean (SD))**	0.98 (0.82)	0.97 (0.82)	0.98 (0.81)	0.96 (0.82)	0.99 (0.82)	1.02 (0.82)		0.87 (0.8)	1 (0.85)
* Missing*	*449 (5.4%)*	*541 (6.8%)*	*403 (7.2%)*	*213 (7.9%)*	*186 (5.1%)*	*56 (5.4%)*		*9 (7.6%)*	*3 (6.0%)*
**Sex of Child = male (%)**									
Male	4399 (53.0%)	4132 (51.7%)	2940 (52.9%)	1363 (50.5%)	1875 (51.9%)	536 (51.5%)		62 (52.1%)	28 (56.0%)
Female	3905 (47.0%)	3855 (48.3%)	2622 (47.1%)	1335 (49.5%)	1739 (48.1%)	505 (48.5%)		57 (47.9%)	22 (44.0%)
* Missing*	*0 (0.0%)*	*0 (0.0%)*	*0 (0.0%)*	*0 (0.0%)*	*0 (0.0%)*	*0 (0.0%)*		*0 (0.0%)*	*0 (0.0%)*
**Mother’s Education (%)**									
No education	2909 (35.0%)	4333 (54.3%)	3621 (65.1%)	2088 (77.4%)	2079 (57.5%)	606 (58.2%)		81 (68.1%)	34 (68.0%)
Primary	1350 (16.3%)	1253 (15.7%)	835 (15.0%)	277 (10.3%)	629 (17.4%)	184 (17.7%)		23 (19.3%)	7 (14.0%)
Secondary	2653 (31.9%)	1867 (23.4%)	926 (16.6%)	274 (10.2%)	717 (19.8%)	207 (19.9%)		13 (10.9%)	8 (16.0%)
Higher	1390 (16.7%)	532 (6.7%)	179 (3.2%)	58 (2.1%)	187 (5.2%)	44 (4.2%)		2 (1.7%)	1 (2.0%)
* Missing*	*2 (0.0%)*	*2 (0.0%)*	*1 (0.0%)*	*1 (0.0%)*	*2 (0.1%)*	*0 (0.0%)*		*0 (0.0%)*	*0 (0.0%)*
**Wealth quintile (%)**									
Poorest	885 (10.7%)	1629 (20.4%)	1585 (28.5%)	1126 (41.7%)	567 (15.7%)	171 (16.4%)		33 (27.7%)	19 (38.0%)
Poorer	1228 (14.8%)	1772 (22.2%)	1332 (23.9%)	770 (28.5%)	619 (17.1%)	174 (16.7%)		34 (28.6%)	15 (30.0%)
Middle	1590 (19.1%)	1825 (22.8%)	1131 (20.3%)	472 (17.5%)	748 (20.7%)	248 (23.8%)		23 (19.3%)	3 (6.0%)
Richer	2100 (25.3%)	1655 (20.7%)	979 (17.6%)	224 (8.3%)	1027 (28.4%)	249 (23.9%)		19 (16.0%)	6 (12.0%)
Richest	2501 (30.1%)	1106 (13.8%)	535 (9.6%)	106 (3.9%)	653 (18.1%)	199 (19.1%)		10 (8.4%)	7 (14.0%)
* Missing*	*0 (0.0%)*	*0 (0.0%)*	*0 (0.0%)*	*0 (0.0%)*	*0 (0.0%)*	*0 (0.0%)*		*0 (0.0%)*	*0 (0.0%)*
**Mother’s height (mean (SD))**	151.88 (7.17)	151.19 (6.36)	150.31 (6.72)	150.94 (6.39)	151.8 (5.7)	151.42 (5.77)		149.79 (4.55)	151.43 (5.86)
*Missing*	*769 (9.3%)*	*536 (6.7%)*	*411 (7.4%)*	*145 (5.4%)*	*427 (11.8%)*	*97 (9.3%)*		*14 (11.8%)*	*4 (8.0%)*
**Urban/ Rural (%)**									
Urban	2662 (32.1%)	1716 (21.5%)	1219 (21.9%)	249 (9.2%)	1291 (35.7%)	364 (35.0%)		28 (23.5%)	13 (26.0%)
Rural	5642 (67.9%)	6271 (78.5%)	4343 (78.1%)	2449 (90.8%)	2323 (64.3%)	677 (65.0%)		91 (76.5%)	37 (74.0%)
* Missing*	*0 (0.0%)*	*0 (0.0%)*	*0 (0.0%)*	*0 (0.0%)*	*0 (0.0%)*	*0 (0.0%)*		*0 (0.0%)*	*0 (0.0%)*
	**NFHS 4**								
	**Hindus (** ***n*** ** = 187,573)**				**Muslim (** ***n*** ** = 40,950)**				
**Variable**	**Other**	**Other Backward Class**	**Scheduled Caste**	**Scheduled Tribe**	**Other**	**Other Backward Class**		**Scheduled Caste**	**Scheduled Tribe**
**N**	**34,082**	**81,525**	**44,258**	**27,708**	**19,260**	**18,316**		**1338**	**2036**
C) NFHS 4									
** Age of child (mean (SD))**	2.03 (1.41)	2.02 (1.41)	2.01 (1.41)	2 (1.42)	2.02 (1.39)	2 (1.41)		2.02 (1.41)	2.01 (1.41)
* Missing*	*1222 (3.6%)*	*3796 (4.7%)*	*2341 (5.3%)*	*1502 (5.4%)*	*852 (4.4%)*	*928 (5.1%)*		*80 (6.0%)*	*86 (4.2%)*
**Sex of Child = male (%)**									
Male	18,087 (53.1%)	42,955 (52.7%)	22,892 (51.7%)	14,166 (51.1%)	9976 (51.8%)	9459 (51.6%)		676 (50.5%)	1060 (52.1%)
Female	15,995 (46.9%)	38,570 (47.3%)	21,366 (48.3%)	13,542 (48.9%)	9284 (48.2%)	8857 (48.4%)		662 (49.5%)	976 (47.9%)
* Missing*	*0 (0.0%)*	*0 (0.0%)*	*0 (0.0%)*	*0 (0.0%)*	*0 (0.0%)*	*0 (0.0%)*		*0 (0.0%)*	*0 (0.0%)*
**Mother’s Education (%)**									
No education	4479 (13.1%)	24,310 (29.8%)	16,133 (36.5%)	13,093 (47.3%)	7048 (36.6%)	8350 (45.6%)		698 (52.2%)	835 (41.0%)
Primary	3555 (10.4%)	10,961 (13.4%)	7247 (16.4%)	4575 (16.5%)	2833 (14.7%)	2997 (16.4%)		229 (17.1%)	180 (8.8%)
Secondary	18,969 (55.7%)	37,905 (46.5%)	18,103 (40.9%)	9155 (33.0%)	8390 (43.6%)	6089 (33.2%)		387 (28.9%)	881 (43.3%)
Higher	7079 (20.8%)	8349 (10.2%)	2775 (6.3%)	885 (3.2%)	989 (5.1%)	880 (4.8%)		24 (1.8%)	140 (6.9%)
* Missing*	*0 (0.0%)*	*0 (0.0%)*	*0 (0.0%)*	*0 (0.0%)*	*0 (0.0%)*	*0 (0.0%)*		*0 (0.0%)*	*0 (0.0%)*
**Wealth quintile (%)**									
Poorest	3363 (9.9%)	20,398 (25.0%)	14,827 (33.5%)	15,277 (55.1%)	4208 (21.8%)	3901 (21.3%)		471 (35.2%)	508 (25.0%)
Poorer	5743 (16.9%)	19,207 (23.6%)	11,458 (25.9%)	6876 (24.8%)	5272 (27.4%)	3927 (21.4%)		319 (23.8%)	608 (29.9%)
Middle	7518 (22.1%)	16,987 (20.8%)	8692 (19.6%)	3283 (11.8%)	3975 (20.6%)	3620 (19.8%)		260 (19.4%)	334 (16.4%)
Richer	7892 (23.2%)	14,443 (17.7%)	5849 (13.2%)	1513 (5.5%)	3361 (17.5%)	3999 (21.8%)		205 (15.3%)	348 (17.1%)
Richest	9566 (28.1%)	10,490 (12.9%)	3432 (7.8%)	759 (2.7%)	2444 (12.7%)	2869 (15.7%)		83 (6.2%)	238 (11.7%)
* Missing*	*0 (0.0%)*	*0 (0.0%)*	*0 (0.0%)*	*0 (0.0%)*	*0 (0.0%)*	*0 (0.0%)*		*0 (0.0%)*	*0 (0.0%)*
**Mother’s height (mean (SD))**	152.84 (6.04)	151.56 (6.14)	150.74 (6.04)	151.02 (5.76)	152.58 (6.34)	152.04 (5.99)		151.83 (5.95)	154.01 (6.12)
*Missing*	*585 (1.7%)*	*833 (1.0%)*	*434 (1.0%)*	*276 (1.0%)*	*288 (1.5%)*	*222 (1.2%)*		*35 (2.6%)*	*19 (0.9%)*
**Urban/ Rural (%)**									
Urban	10,917 (32.0%)	17,957 (22.0%)	9107 (20.6%)	2353 (8.5%)	5493 (28.5%)	7666 (41.9%)		383 (28.6%)	497 (24.4%)
Rural	23,165 (68.0%)	63,568 (78.0%)	35,151 (79.4%)	25,355 (91.5%)	13,767 (71.5%)	10,650 (58.1%)		955 (71.4%)	1539 (75.6%)
* Missing*	*0 (0.0%)*	*0 (0.0%)*	*0 (0.0%)*	*0 (0.0%)*	*0 (0.0%)*	*0 (0.0%)*		*0 (0.0%)*	*0 (0.0%)*
	**NFHS 3**								
	**Hindu (** ***n*** ** = 35,499)**				**Muslim (** ***n*** ** = 8595)**				
**Variable**	**Other**	**Other Backward Class**	**Scheduled Caste**	**Scheduled Tribe**	**Other**	**Other Backward Class**		**Scheduled Caste**	**Scheduled Tribe**
**N**	**10,453**	**13,288**	**8226**	**3532**	**5183**	**2994**		**253**	**165**
D) NFHS 3									
** Age of child (mean (SD))**	2.02 (1.41)	2.03 (1.42)	2.02 (1.41)	2.01 (1.43)	2.02 (1.41)	2.06 (1.41)		1.9 (1.38)	1.9 (1.42)
* Missing*	*460 (4.4%)*	*770 (5.8%)*	*567 (6.9%)*	*266 (7.5%)*	*273 (5.3%)*	*154 (5.1%)*		*19 (7.5%)*	*5 (3.0%)*
**Sex of Child = male (%)**									
Male	5543 (53.0%)	6948 (52.3%)	4201 (51.1%)	1845 (52.2%)	2690 (51.9%)	1536 (51.3%)		133 (52.6%)	77 (46.7%)
Female	4910 (47.0%)	6340 (47.7%)	4025 (48.9%)	1687 (47.8%)	2493 (48.1%)	1458 (48.7%)		120 (47.4%)	88 (53.3%)
* Missing*	*0 (0.0%)*	*0 (0.0%)*	*0 (0.0%)*	*0 (0.0%)*	*0 (0.0%)*	*0 (0.0%)*		*0 (0.0%)*	*0 (0.0%)*
**Mother’s Education (%)**									
No education	2126 (20.3%)	5788 (43.6%)	4262 (51.8%)	2398 (67.9%)	2323 (44.8%)	1798 (60.1%)		160 (63.2%)	117 (70.9%)
Primary	1255 (12.0%)	1962 (14.8%)	1300 (15.8%)	428 (12.1%)	810 (15.6%)	383 (12.8%)		32 (12.6%)	12 (7.3%)
Secondary	5146 (49.2%)	4734 (35.6%)	2417 (29.4%)	661 (18.7%)	1836 (35.4%)	746 (24.9%)		59 (23.3%)	36 (21.8%)
Higher	1925 (18.4%)	804 (6.1%)	247 (3.0%)	45 (1.3%)	214 (4.1%)	67 (2.2%)		2 (0.8%)	0 (0.0%)
* Missing*	*1 (0.0%)*	*0 (0.0%)*	*0 (0.0%)*	*0 (0.0%)*	*0 (0.0%)*	*0 (0.0%)*		*0 (0.0%)*	*0 (0.0%)*
**Wealth quintile (%)**									
Poorest	647 (6.2%)	2445 (18.4%)	2077 (25.2%)	1844 (52.2%)	837 (16.1%)	489 (16.3%)		53 (20.9%)	30 (18.2%)
Poorer	1133 (10.8%)	2845 (21.4%)	1900 (23.1%)	746 (21.1%)	912 (17.6%)	525 (17.5%)		43 (17.0%)	58 (35.2%)
Middle	1950 (18.7%)	2861 (21.5%)	1746 (21.2%)	471 (13.3%)	1068 (20.6%)	711 (23.7%)		62 (24.5%)	34 (20.6%)
Richer	2612 (25.0%)	2799 (21.1%)	1619 (19.7%)	300 (8.5%)	1263 (24.4%)	817 (27.3%)		65 (25.7%)	36 (21.8%)
Richest	4111 (39.3%)	2338 (17.6%)	884 (10.7%)	171 (4.8%)	1103 (21.3%)	452 (15.1%)		30 (11.9%)	7 (4.2%)
*Missing*	*0 (0.0%)*	*0 (0.0%)*	*0 (0.0%)*	*0 (0.0%)*	*0 (0.0%)*	*0 (0.0%)*		*0 (0.0%)*	*0 (0.0%)*
**Mother’s height (mean (SD))**	152.76 (6)	151.66 (5.83)	150.88 (5.64)	151.36 (5.73)	152.28 (5.78)	152.07 (5.73)		151.21 (6.05)	154.7 (5.73)
*Missing*	*480 (4.6%)*	*445 (3.3%)*	*357 (4.3%)*	*116 (3.3%)*	*332 (6.4%)*	*219 (7.3%)*		*13 (5.1%)*	*2 (1.2%)*
**Urban/ Rural (%)**									
Urban	5049 (48.3%)	4413 (33.2%)	2848 (34.6%)	542 (15.3%)	2448 (47.2%)	1545 (51.6%)		124 (49.0%)	16 (9.7%)
Rural	5404 (51.7%)	8875 (66.8%)	5378 (65.4%)	2990 (84.7%)	2735 (52.8%)	1449 (48.4%)		129 (51.0%)	149 (90.3%)
* Missing*	*0 (0.0%)*	*0 (0.0%)*	*0 (0.0%)*	*0 (0.0%)*	*0 (0.0%)*	*0 (0.0%)*		*0 (0.0%)*	*0 (0.0%)*
	**NFHS 5**								
	**Hindu (** ***n*** ** = 171,055)**				**Muslim (** ***n*** ** = 33,522)**				
**Variable**	**Other**	**Other Backward Class**	**Scheduled Caste**	**Scheduled Tribe**	**Other**		**Other Backward Class**	**Scheduled Caste**	**Scheduled Tribe**
**N**	**29,167**	**73,351**	**42,938**	**25,599**	**16,651**		**14,293**	**1256**	**1322**
E) NFHS 5									
** Age of child (mean (SD))**	2.05 (1.42)	2.02 (1.43)	2 (1.43)	2 (1.43)	2.06 (1.42)		2.01 (1.43)	2.05 (1.42)	2.09 (1.42)
* Missing*	*841 (2.9%)*	*2765 (3.8%)*	*1959 (4.6%)*	*1171 (4.6%)*	*537 (3.2%)*		*515 (3.6%)*	*33 (2.6%)*	*40 (3.0%)*
**Sex of Child = male (%)**									
Male	15,382 (52.7%)	38,246 (52.1%)	22,256 (51.8%)	13,105 (51.2%)	8529 (51.2%)		7390 (51.7%)	640 (51.0%)	655 (49.5%)
Female	13,785 (47.3%)	35,105 (47.9%)	20,682 (48.2%)	12,494 (48.8%)	8122 (48.8%)		6903 (48.3%)	616 (49.0%)	667 (50.5%)
* Missing*	*0 (0.0%)*	*0 (0.0%)*	*0 (0.0%)*	*0 (0.0%)*	*0 (0.0%)*		*0 (0.0%)*	*0 (0.0%)*	*0 (0.0%)*
**Mother’s Education (%)**									
No education	2688 (9.2%)	14,274 (19.5%)	10,933 (25.5%)	9013 (35.2%)	4102 (24.6%)		4726 (33.1%)	504 (40.1%)	389 (29.4%)
Primary	2445 (8.4%)	8074 (11.0%)	6227 (14.5%)	3801 (14.8%)	2430 (14.6%)		2051 (14.3%)	190 (15.1%)	137 (10.4%)
Secondary	16,146 (55.4%)	38,923 (53.1%)	21,358 (49.7%)	11,380 (44.5%)	8856 (53.2%)		6244 (43.7%)	518 (41.2%)	672 (50.8%)
Higher	7888 (27.0%)	12,080 (16.5%)	4420 (10.3%)	1405 (5.5%)	1263 (7.6%)		1272 (8.9%)	44 (3.5%)	124 (9.4%)
* Missing*	*0 (0.0%)*	*0 (0.0%)*	*0 (0.0%)*	*0 (0.0%)*	*0 (0.0%)*		*0 (0.0%)*	*0 (0.0%)*	*0 (0.0%)*
**Wealth quintile (%)**									
Poorest	3057 (10.5%)	16,087 (21.9%)	13,605 (31.7%)	13,847 (54.1%)	4628 (27.8%)		2626 (18.4%)	391 (31.1%)	376 (28.4%)
Poorer	4983 (17.1%)	17,465 (23.8%)	11,143 (26.0%)	6278 (24.5%)	3966 (23.8%)		2908 (20.3%)	342 (27.2%)	300 (22.7%)
Middle	5717 (19.6%)	16,181 (22.1%)	8460 (19.7%)	3223 (12.6%)	3144 (18.9%)		2922 (20.4%)	202 (16.1%)	245 (18.5%)
Richer	6811 (23.4%)	14,242 (19.4%)	6044 (14.1%)	1573 (6.1%)	2810 (16.9%)		3259 (22.8%)	208 (16.6%)	266 (20.1%)
Richest	8599 (29.5%)	9376 (12.8%)	3686 (8.6%)	678 (2.6%)	2103 (12.6%)		2578 (18.0%)	113 (9.0%)	135 (10.2%)
* Missing*	*0 (0.0%)*	*0 (0.0%)*	*0 (0.0%)*	*0 (0.0%)*	*0 (0.0%)*		*0 (0.0%)*	*0 (0.0%)*	*0 (0.0%)*
**Mother's height (mean (SD))**	153.07 (6.37)	151.78 (6.35)	150.85 (6.23)	151.25 (5.95)	152.54 (6.6)		152.64 (6.27)	151.95 (7.39)	154.09 (7.41)
* Missing*	*855 (2.9%)*	*1776 (2.4%)*	*1025 (2.4%)*	*461 (1.8%)*	*529 (3.2%)*		*722 (5.1%)*	*62 (4.9%)*	*22 (1.7%)*
**Urban/ Rural (%)**									
Urban	8690 (29.8%)	14,100 (19.2%)	7874 (18.3%)	1934 (7.6%)	4196 (25.2%)		4954 (34.7%)	324 (25.8%)	311 (23.5%)
Rural	20,477 (70.2%)	59,251 (80.8%)	35,064 (81.7%)	23,665 (92.4%)	12,455 (74.8%)		9339 (65.3%)	932 (74.2%)	1011 (76.5%)
* Missing*	*0 (0.0%)*	*0 (0.0%)*	*0 (0.0%)*	*0 (0.0%)*	*0 (0.0%)*		*0 (0.0%)*	*0 (0.0%)*	*0 (0.0%)*

^a^Other Backward Class was not legally recognized as a deprived community at the time of data collection

### Distribution of outcomes in the sample

27% Hindu Other castes, 35% Hindu OBCs, 40% Hindu SCs and 40% Hindu STs were stunted, compared to 35% Muslim Other castes, 37% Muslim OBC, 41% Muslim Dalits and 33% Muslim STs (Table 2). Hindus across castes/tribes improved on all three anthropometric outcomes between NFHS 4 and 5, but for underweight, only Muslim OBCs saw a reduction in prevalence (Table 2). Prevalence of wasting increased in Muslim Other castes and STs and remained consistent among Muslim OBCs (Table 2).

**Table 2:** Distribution of outcomes among social strata of religion- caste and religion-tribe, in Hindu-Muslim children and their differentials across NFHS waves nationally

	**NFHS 1**							
	**Hindu (** ***n*** ** = 37,231)**				**Muslim (** ***n*** ** = 6594)**			
**Variable**	**Other**	**Other Backward Class**	**Scheduled Caste**	**Scheduled Tribe**	**Other**	**Other Backward Class**	**Scheduled Caste**	**Scheduled Tribe**
**n**	**27,467**	**NA**	**5903**	**3861**	**6351**	**NA**	**201**	**42**
A) NFHS 1^a^								
** HAZ (mean (SD))**	-1.9 (1.71)	NA	-2.17 (1.72)	-1.94 (1.93)	-2.09 (1.69)	NA	-1.97 (1.82)	-2.5 (1.18)
* Missing*	*12,300 (44.8%)*	*NA*	*2784 (47.2%)*	*2263 (58.6%)*	*2874 (45.3%)*	*NA*	*98 (48.8%)*	*25 (59.5%)*
** WAZ (mean (SD))**	-1.89 (1.29)	NA	-2.1 (1.31)	-2.17 (1.49)	-2.03 (1.26)	NA	-1.97 (1.18)	-1.9 (1.04)
* Missing*	7085 (25.8%)	NA	1680 (28.5%)	1113 (28.8%)	1770 (27.9%)	NA	77 (38.3%)	8 (19.0%)
** WHZ (mean (SD))**	-0.9 (1.16)	NA	-0.97 (1.19)	-1.05 (1.34)	-0.91 (1.14)	NA	-0.96 (1.17)	-0.79 (0.82)
* Missing*	*12,248 (44.6%)*	*NA*	*2765 (46.8%)*	*2250 (58.3%)*	*2844 (44.8%)*	*NA*	*98 (48.8%)*	*25 (59.5%)*
**Stunting (%)**		NA				NA		
Yes	7265 (26.4%)	NA	1768 (30.0%)	830 (21.5%)	1827 (28.8%)	NA	53 (26.4%)	11 (26.2%)
No	7902 (28.8%)	NA	1351 (22.9%)	768 (19.9%)	1650 (26.0%)	NA	50 (24.9%)	6 (14.3%)
* Missing*	12,300 (44.8%)	*NA*	*2784 (47.2%)*	*2263 (58.6%)*	*2874 (45.3%)*	*NA*	*98 (48.8%)*	*25 (59.5%)*
**Underweight (%)**		NA				NA		
Yes	10,104 (36.8%)	NA	2411 (40.8%)	1629 (42.2%)	2477 (39.0%)	NA	56 (27.9%)	17 (40.5%)
No	10,278 (37.4%)	NA	1812 (30.7%)	1119 (29.0%)	2104 (33.1%)	NA	68 (33.8%)	17 (40.5%)
* Missing*	7085 (25.8%)	NA	1680 (28.5%)	1113 (28.8%)	1770 (27.9%)	NA	77 (38.3%)	8 (19.0%)
**Wasting (%)**		NA				NA		
Yes	2406 (8.8%)	NA	550 (9.3%)	359 (9.3%)	546 (8.6%)	NA	16 (8.0%)	2 (4.8%)
No	12,813 (46.6%)	NA	2588 (43.8%)	1252 (32.4%)	2961 (46.6%)	NA	87 (43.3%)	15 (35.7%)
* Missing*	*12,248 (44.6%)*	*NA*	*2765 (46.8%)*	*2250 (58.3%)*	*2844 (44.8%)*	*NA*	*98 (48.8%)*	*25 (59.5%)*
	**NFHS 2**							
	**Hindu (** ***n*** ** = 24,551)**				**Muslim (** ***n*** ** = 4824)**			
**Variable**	**Other**	**Other Backward Class**	**Scheduled Caste**	**Scheduled Tribe**	**Other**	**Other Backward Class**	**Scheduled Caste**	**Scheduled Tribe**
**N**	**8304**	**7987**	**5562**	**2698**	**3614**	**1041**	**119**	**50**
B) NFHS 2								
** HAZ (mean (SD))**	-1.55 (1.57)	-1.79 (1.68)	-2.02 (1.67)	-2.02 (1.78)	-1.75 (1.73)	-1.88 (1.75)	-2.3 (1.44)	-1.83 (1.88)
* Missing*	*1969 (23.7%)*	*1841 (23.0%)*	*1377 (24.8%)*	*721 (26.7%)*	*1067 (29.5%)*	*270 (25.9%)*	*43 (36.1%)*	*12 (24.0%)*
**WAZ (mean (SD))**	-1.53 (1.29)	-1.85 (1.32)	-1.95 (1.33)	-2.1 (1.4)	-1.69 (1.34)	-1.83 (1.35)	-2.08 (1.27)	-1.39 (2)
* Missing*	1969 (23.7%)	1841 (23.0%)	1377 (24.8%)	721 (26.7%)	1067 (29.5%)	270 (25.9%)	43 (36.1%)	12 (24.0%)
**WHZ (mean (SD))**	-0.7 (1.21)	-0.92 (1.2)	-0.87 (1.26)	-1.05 (1.3)	-0.74 (1.26)	-0.81 (1.29)	-0.73 (1.35)	-0.3 (1.77)
* Missing*	*1950 (23.5%)*	*1828 (22.9%)*	*1347 (24.2%)*	*692 (25.6%)*	*1040 (28.8%)*	*266 (25.6%)*	*40 (33.6%)*	*12 (24.0%)*
**Stunting (%)**								
Yes	2402 (28.9%)	2736 (34.3%)	2144 (38.5%)	1043 (38.7%)	1116 (30.9%)	366 (35.2%)	40 (33.6%)	19 (38.0%)
No	3933 (47.4%)	3410 (42.7%)	2041 (36.7%)	934 (34.6%)	1431 (39.6%)	405 (38.9%)	36 (30.3%)	19 (38.0%)
* Missing*	*1969 (23.7%)*	*1841 (23.0%)*	*1377 (24.8%)*	*721 (26.7%)*	*1067 (29.5%)*	*270 (25.9%)*	*43 (36.1%)*	*12 (24.0%)*
**Underweight (%)**								
Yes	2335 (28.1%)	2946 (36.9%)	2185 (39.3%)	1116 (41.4%)	1101 (30.5%)	370 (35.5%)	40 (33.6%)	16 (32.0%)
No	4000 (48.2%)	3200 (40.1%)	2000 (36.0%)	861 (31.9%)	1446 (40.0%)	401 (38.5%)	36 (30.3%)	22 (44.0%)
* Missing*	1969 (23.7%)	1841 (23.0%)	1377 (24.8%)	721 (26.7%)	1067 (29.5%)	270 (25.9%)	43 (36.1%)	12 (24.0%)
**Wasting (%)**								
Yes	770 (9.3%)	1050 (13.1%)	692 (12.4%)	445 (16.5%)	348 (9.6%)	120 (11.5%)	10 (8.4%)	7 (14.0%)
No	5584 (67.2%)	5109 (64.0%)	3523 (63.3%)	1561 (57.9%)	2226 (61.6%)	655 (62.9%)	69 (58.0%)	31 (62.0%)
* Missing*	*1950 (23.5%)*	*1828 (22.9%)*	*1347 (24.2%)*	*692 (25.6%)*	*1040 (28.8%)*	*266 (25.6%)*	*40 (33.6%)*	*12 (24.0%)*
	**NFHS 3**							
	**Hindu (** ***n*** ** = 35,499)**				**Muslim (** ***n*** ** = 8595)**			
**Variable**	**Other**	**Other Backward Class**	**Scheduled Caste**	**Scheduled Tribe**	**Other**	**Other Backward Class**	**Scheduled Caste**	**Scheduled Tribe**
**n**	**10,453**	**13,288**	**8226**	**3532**	**5183**	**2994**	**253**	**165**
C) NFHS 3								
** HAZ (mean (SD))**	-1.36 (1.56)	-1.79 (1.64)	-1.97 (1.64)	-2.01 (1.7)	-1.71 (1.66)	-1.97 (1.74)	-1.91 (1.72)	-1.4 (2)
* Missing*	*1955 (18.7%)*	*2481 (18.7%)*	*1682 (20.4%)*	*740 (21.0%)*	*1111 (21.4%)*	*675 (22.5%)*	*68 (26.9%)*	*27 (16.4%)*
**WAZ (mean (SD))**	-1.32 (1.22)	-1.73 (1.2)	-1.84 (1.21)	-2.07 (1.25)	-1.57 (1.23)	-1.79 (1.23)	-1.8 (1.24)	-1.47 (1.39)
* Missing*	1955 (18.7%)	2481 (18.7%)	1682 (20.4%)	740 (21.0%)	1111 (21.4%)	675 (22.5%)	68 (26.9%)	27 (16.4%)
**WHZ (mean (SD))**	-0.78 (1.3)	-1.02 (1.28)	-1.02 (1.29)	-1.3 (1.3)	-0.84 (1.31)	-0.93 (1.3)	-0.97 (1.46)	-0.94 (1.43)
* Missing*	*1955 (18.7%)*	*2481 (18.7%)*	*1682 (20.4%)*	*740 (21.0%)*	*1111 (21.4%)*	*675 (22.5%)*	*68 (26.9%)*	*27 (16.4%)*
**Stunting (%)**								
Yes	2910 (27.8%)	4959 (37.3%)	3361 (40.9%)	1442 (40.8%)	1793 (34.6%)	1154 (38.5%)	99 (39.1%)	55 (33.3%)
No	5588 (53.5%)	5848 (44.0%)	3183 (38.7%)	1350 (38.2%)	2279 (44.0%)	1165 (38.9%)	86 (34.0%)	83 (50.3%)
* Missing*	*1955 (18.7%)*	*2481 (18.7%)*	*1682 (20.4%)*	*740 (21.0%)*	*1111 (21.4%)*	*675 (22.5%)*	*68 (26.9%)*	*27 (16.4%)*
**Underweight (%)**								
Yes	2383 (22.8%)	4428 (33.3%)	2977 (36.2%)	1492 (42.2%)	1439 (27.8%)	988 (33.0%)	87 (34.4%)	50 (30.3%)
No	6115 (58.5%)	6379 (48.0%)	3567 (43.4%)	1300 (36.8%)	2633 (50.8%)	1331 (44.5%)	98 (38.7%)	88 (53.3%)
* Missing*	1955 (18.7%)	2481 (18.7%)	1682 (20.4%)	740 (21.0%)	1111 (21.4%)	675 (22.5%)	68 (26.9%)	27 (16.4%)
**Wasting (%)**								
Yes	1291 (12.4%)	2132 (16.0%)	1330 (16.2%)	757 (21.4%)	685 (13.2%)	439 (14.7%)	41 (16.2%)	31 (18.8%)
No	7207 (68.9%)	8675 (65.3%)	5214 (63.4%)	2035 (57.6%)	3387 (65.3%)	1880 (62.8%)	144 (56.9%)	107 (64.8%)
* Missing*	*1955 (18.7%)*	*2481 (18.7%)*	*1682 (20.4%)*	*740 (21.0%)*	*1111 (21.4%)*	*675 (22.5%)*	*68 (26.9%)*	*27 (16.4%)*
	**NFHS 4**							
	**Hindu (** ***n*** ** = 187,573)**				**Muslim (** ***n*** ** = 40,950)**			
**Variable**	**Other**	**Other Backward Class**	**Scheduled Caste**	**Scheduled Tribe**	**Other**	**Other Backward Class**	**Scheduled Caste**	**Scheduled Tribe**
**n**	**34,082**	**81,525**	**44,258**	**27,708**	**19,260**	**18,316**	**1338**	**2036**
D) NFHS 4								
** HAZ (mean (SD))**	-1.14 (1.63)	-1.52 (1.65)	-1.69 (1.65)	-1.7 (1.72)	-1.41 (1.73)	-1.64 (1.66)	-1.69 (1.66)	-1.35 (1.74)
* Missing*	*4276 (12.5%)*	*10,326 (12.7%)*	*5830 (13.2%)*	*4052 (14.6%)*	*2759 (14.3%)*	*2412 (13.2%)*	*259 (19.4%)*	*279 (13.7%)*
**WAZ (mean (SD))**	-1.24 (1.2)	-1.6 (1.18)	-1.72 (1.18)	-1.86 (1.2)	-1.38 (1.23)	-1.63 (1.18)	-1.62 (1.19)	-1.28 (1.26)
* Missing*	4276 (12.5%)	10,326 (12.7%)	5830 (13.2%)	4052 (14.6%)	2759 (14.3%)	2412 (13.2%)	259 (19.4%)	279 (13.7%)
**WHZ (mean (SD))**	-0.84 (1.4)	-1.04 (1.35)	-1.07 (1.35)	-1.26 (1.42)	-0.81 (1.38)	-1 (1.33)	-0.93 (1.35)	-0.72 (1.46)
* Missing*	*4276 (12.5%)*	*10,326 (12.7%)*	*5830 (13.2%)*	*4052 (14.6%)*	*2759 (14.3%)*	*2412 (13.2%)*	*259 (19.4%)*	*279 (13.7%)*
**Stunting (%)**								
Yes	8619 (25.3%)	28,045 (34.4%)	16,976 (38.4%)	10,506 (37.9%)	6156 (32.0%)	6790 (37.1%)	501 (37.4%)	624 (30.6%)
No	21,187 (62.2%)	43,154 (52.9%)	21,452 (48.5%)	13,150 (47.5%)	10,345 (53.7%)	9114 (49.8%)	578 (43.2%)	1133 (55.6%)
* Missing*	*4276 (12.5%)*	*10,326 (12.7%)*	*5830 (13.2%)*	*4052 (14.6%)*	*2759 (14.3%)*	*2412 (13.2%)*	*259 (19.4%)*	*279 (13.7%)*
**Underweight (%)**								
Yes	7754 (22.8%)	26,153 (32.1%)	15,606 (35.3%)	10,945 (39.5%)	4997 (25.9%)	5988 (32.7%)	416 (31.1%)	483 (23.7%)
No	22,052 (64.7%)	45,046 (55.3%)	22,822 (51.6%)	12,711 (45.9%)	11,504 (59.7%)	9916 (54.1%)	663 (49.6%)	1274 (62.6%)
* Missing*	4276 (12.5%)	10,326 (12.7%)	5830 (13.2%)	4052 (14.6%)	2759 (14.3%)	2412 (13.2%)	259 (19.4%)	279 (13.7%)
**Wasting (%)**								
Yes	5423 (15.9%)	15,194 (18.6%)	8464 (19.1%)	6744 (24.3%)	2820 (14.6%)	3176 (17.3%)	213 (15.9%)	297 (14.6%)
No	24,383 (71.5%)	56,005 (68.7%)	29,964 (67.7%)	16,912 (61.0%)	13,681 (71.0%)	12,728 (69.5%)	866 (64.7%)	1460 (71.7%)
* Missing*	*4276 (12.5%)*	*10,326 (12.7%)*	*5830 (13.2%)*	*4052 (14.6%)*	*2759 (14.3%)*	*2412 (13.2%)*	*259 (19.4%)*	*279 (13.7%)*
	**NFHS 5**							
	**Hindu (** ***n*** ** = 171,055)**				**Muslim (** ***n*** ** = 33,522)**			
**Variable**	**Other**	**Other Backward Class**	**Scheduled Caste**	**Scheduled Tribe**	**Other**	**Other Backward Class**	**Scheduled Caste**	**Scheduled Tribe**
**n**	**29,167**	**73,351**	**42,938**	**25,599**	**16,651**	**14,293**	**1256**	**1322**
E) NFHS 5								
** HAZ (mean (SD))**	-1.04 (1.76)	-1.32 (1.78)	-1.48 (1.8)	-1.49 (1.87)	-1.2 (1.98)	-1.33 (1.9)	-1.38 (1.96)	-0.96 (2.15)
* Missing*	*3270 (11.2%)*	*8551 (11.7%)*	*5211 (12.1%)*	*3014 (11.8%)*	*1947 (11.7%)*	*2054 (14.4%)*	*166 (13.2%)*	*145 (11.0%)*
**WAZ (mean (SD))**	-1.12 (1.35)	-1.45 (1.28)	-1.55 (1.3)	-1.68 (1.35)	-1.35 (1.44)	-1.48 (1.36)	-1.49 (1.37)	-1.09 (1.6)
* Missing*	2725 (9.3%)	7229 (9.9%)	4361 (10.2%)	2484 (9.7%)	1538 (9.2%)	1761 (12.3%)	140 (11.1%)	108 (8.2%)
**WHZ (mean (SD))**	-0.64 (1.52)	-0.86 (1.48)	-0.87 (1.5)	-0.99 (1.54)	-0.8 (1.66)	-0.85 (1.51)	-0.84 (1.61)	-0.52 (1.91)
* Missing*	*3741 (12.8%)*	*9835 (13.4%)*	*5893 (13.7%)*	*3644 (14.2%)*	*2376 (14.3%)*	*2399 (16.8%)*	*199 (15.8%)*	*190 (14.4%)*
**Stunting (%)**								
Yes	7122 (24.4%)	22,958 (31.3%)	15,004 (34.9%)	9206 (36.0%)	5164 (31.0%)	4516 (31.6%)	443 (35.3%)	390 (29.5%)
No	18,775 (64.4%)	41,842 (57.0%)	22,723 (52.9%)	13,379 (52.3%)	9540 (57.3%)	7723 (54.0%)	647 (51.5%)	787 (59.5%)
* Missing*	*3270 (11.2%)*	*8551 (11.7%)*	*5211 (12.1%)*	*3014 (11.8%)*	*1947 (11.7%)*	*2054 (14.4%)*	*166 (13.2%)*	*145 (11.0%)*
**Underweight (%)**								
Yes	6233 (21.4%)	21,052 (28.7%)	13,535 (31.5%)	9163 (35.8%)	4736 (28.4%)	4186 (29.3%)	398 (31.7%)	345 (26.1%)
No	20,209 (69.3%)	45,070 (61.4%)	25,042 (58.3%)	13,952 (54.5%)	10,377 (62.3%)	8346 (58.4%)	718 (57.2%)	869 (65.7%)
* Missing*	2725 (9.3%)	7229 (9.9%)	4361 (10.2%)	2484 (9.7%)	1538 (9.2%)	1761 (12.3%)	140 (11.1%)	108 (8.2%)
**Wasting (%)**								
Yes	4009 (13.7%)	12,167 (16.6%)	7238 (16.9%)	5041 (19.7%)	2970 (17.8%)	2410 (16.9%)	214 (17.0%)	233 (17.6%)
No	21,417 (73.4%)	51,349 (70.0%)	29,807 (69.4%)	16,914 (66.1%)	11,305 (67.9%)	9484 (66.4%)	843 (67.1%)	899 (68.0%)
* Missing*	*3741 (12.8%)*	*9835 (13.4%)*	*5893 (13.7%)*	*3644 (14.2%)*	*2376 (14.3%)*	*2399 (16.8%)*	*199 (15.8%)*	*190 (14.4%)*

^a^Other Backward Class was not legally recognized as a deprived community at the time of data collection

### Estimates of child growth outcomes by social strata of religion-caste and religion-tribe identities

**Fig. 1:**
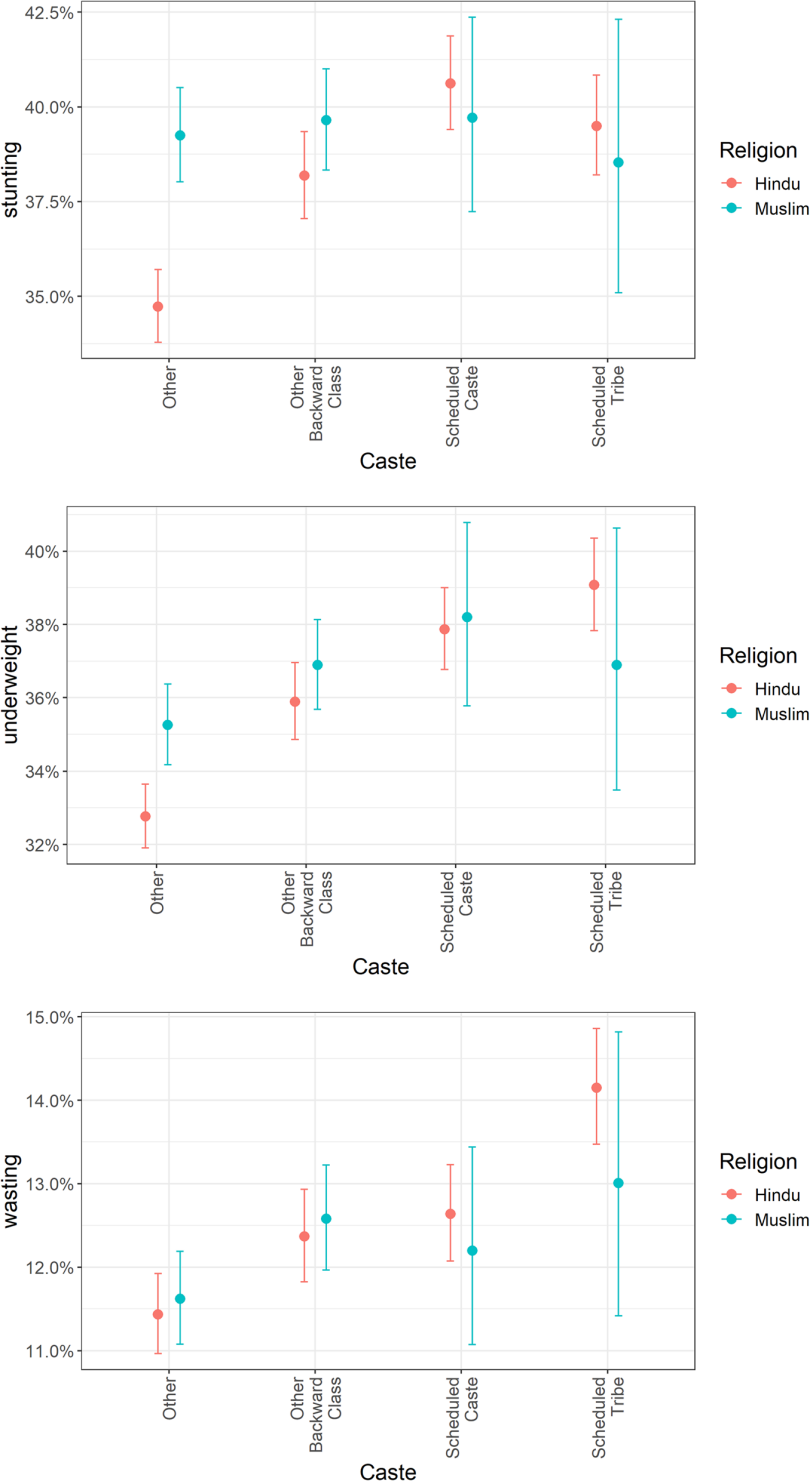
Predicted prevalence of child growth outcomes within strata of religion and caste/tribal identities in adjusted models. These estimates are adjusted for household wealth, mother's education, mother’s height (for stunting and wasting), mother’s weight (for underweight and wasting), child’s age, child's sex, urbanicity. We also use state and district fixed effects, and included each NFHS survey wave as a fixed effect to control for all state invariant factors that may vary over time

In adjusted models, Hindu Other (forward) castes had the lowest prevalence of growth failures at 34.7% (95%CI: 33.8, 5.7) for stunting, 32.8% (95%CI: 31.9, 33.7) for underweight and 11.4% (95%CI: 11,11.9) for wasting (Table 3A). The prevalence of stunting and underweight for Muslim Other (forward) castes was higher than that of Hindu forward castes, and higher or comparable to that of Hindu deprived castes (Hindu SCs and Hindu OBCs). Prevalence of stunting in Muslim Other (forward) Castes was 39.2% (95%CI: 38, 40.5), compared to Hindu SCs’ 39.5% (95%CI: 38.2, 40.8), and Hindu OBCs’ 38.2% (95%CI: 37.1, 39.3) (Table 3A). Prevalence of underweight in Muslim Other (forward) castes was 35.3% (95%CI: 34.2, 36.4), compared to Hindu OBCs’ 35.9% (95%CI: 34.9, 37) and Hindu SCs’ 39.1% (95%CI: 37.8, 40.4) (Table 3B). Thus, in the prevalence of stunting and underweight, Muslim forward castes, Muslim OBCs and Muslim SCs did worse than their Hindu counterparts from each social group (Fig. 1). However, in the prevalence of wasting, Muslim forward castes [11.6% (95%CI: 11.1,12.2)] were closer to Hindu advantaged castes [ 11.4% (95%CI: 11,11.9)], compared to Hindu OBCs’ 12.4% (95%CI: 11.8,12.9) and Hindu SCs’ 14.1% (95%CI: 13.5,14.9) (Table 3C).

**Table 3:** Predicted percentage prevalence of stunting, underweight and wasting within strata of religion across waves of National Family Health Surveys. These estimates are adjusted for household wealth, mother's education, mother’s height (for stunting and wasting), mother’s weight (for underweight and wasting), child’s age, child's sex, urbanicity. We also use state and district fixed effects, and included each NFHS survey wave as a fixed effect to control for all state invariant factors that may vary over time

A) Stunting			
**NFHS**	**Caste**	**Hindu** **% Prevalence (95% CI)**	**Muslim** **% Prevalence (95% CI)**
NFHS 1—5	Other	34.7 [33.8, 35.7]	39.2 [38, 40.5]
	Other Backward Class	38.2 [37.1, 39.3]	39.6 [38.3, 41]
	Scheduled Caste	39.5 [38.2, 40.8]	38.5 [35.1, 42.3]
	Scheduled Tribe	40.6 [39.4, 41.9]	39.7 [37.2, 42.4]
B) Underweight			
**NFHS**	**Caste**	**Hindu** **% Prevalence (95% CI)**	**Muslim** **% Prevalence (95% CI)**
NFHS 1	Other	46.3 [42.4, 50.5]	47.9 [43.6, 52.6]
	Other Backward Class		
	Scheduled Caste	49.5 [44.9, 54.5]	48 [33.6, 68.5]
	Scheduled Tribe	46.8 [41.9, 52.3]	44.2 [16.6, 117.8]
NFHS 2	Other	34.5 [31, 38.5]	39.4 [35.1, 44.1]
	Other Backward Class	36.8 [30, 38]	40.2 [34.7, 46.5]
	Scheduled Caste	38.9 [34.8, 43.5]	40.8 [29.8, 55.9]
	Scheduled Tribe	39.4 [34.8, 44.6]	37 [23.4, 58.7]
NFHS 3	Other	32.5 [29.9, 35.2]	36.6 [33.6, 39.8]
	Other Backward Class	35.2 [32.6, 38]	35.3 [32.3, 38.6]
	Scheduled Caste	37.2 [34.3, 40.3]	41 [33, 50.9]
	Scheduled Tribe	35.9 [32.7, 39.3]	33 [20.9, 52]
NFHS 4	Other	25.6 [24.6, 26.6]	30.6 [29.3, 32]
	Other Backward Class	28.5 [27.5, 29.5]	30.1 [28.8, 31.3]
	Scheduled Caste	29.7 [28.6, 30.9]	28.7 [26.1, 31.6]
	Scheduled Tribe	29.5 [28.2, 30.8]	31.6 [27.9, 35.8]
NFHS 5	Other	24.3 [23.4, 25.3]	28 [26.7, 29.3]
	Other Backward Class	26.9 [25.9, 27.9]	29.2 [28, 30.5]
	Scheduled Caste	28.3 [27.2, 29.4]	30.1 [27.4, 33.1]
	Scheduled Tribe	27.5 [26.3, 28.8]	26.2 [22.5, 30.5]
**NFHS**	**Caste**	**Hindu** **Prevalence [95% CI]**	**Muslim** **Prevalence [95% CI]**
NFHS 1—5	Other	32.8 [31.9, 33.7]	35.3 [34.2, 36.4]
	Other Backward Class	35.9 [34.9, 37]	36.9 [35.7, 38.1]
	Scheduled Caste	39.1 [37.8, 40.4]	36.9 [33.5, 40.6]
	Scheduled Tribe	37.9 [36.8, 39]	38.2 [35.8, 40.8]
**NFHS**	**Caste**	**Hindu** **Prevalence [95% CI]**	Muslim **Prevalence [95% CI]**
NFHS 1	Other	44.1 [40.9, 47.5]	45.8 [42.3, 49.6]
	Other Backward Class		
	Scheduled Caste	46 [42.3, 49.9]	44.5 [32.5, 60.8]
	Scheduled Tribe	44.8 [40.9, 49.1]	35.7 [16.8, 75.9]
NFHS 2	Other	27.5 [24.7, 30.6]	32.3 [28.8, 36.2]
	Other Backward Class	31.1 [27.9, 34.7]	32.9 [28.5, 38]
	Scheduled Caste	32.2 [28.8, 36]	32.1 [23.2, 44.3]
	Scheduled Tribe	32.7 [28.9, 37]	28.2 [17.3, 45.9]
NFHS 3	Other	22.6 [20.6, 24.7]	24 [21.8, 26.4]
	Other Backward Class	24.7 [22.6, 26.8]	24.4 [22.1, 26.9]
	Scheduled Caste	26.7 [24.5, 29.2]	28.6 [22.6, 36.2]
	Scheduled Tribe	27.4 [24.8, 30.2]	28 [17.3, 45.6]
NFHS 4	Other	21.6 [20.8, 22.5]	23.7 [22.6, 24.8]
	Other Backward Class	23.7 [22.8, 24.6]	24.6 [23.6, 25.7]
	Scheduled Caste	24.6 [23.7, 25.6]	24.9 [22.6, 27.4]
	Scheduled Tribe	26.3 [25.1, 27.5]	25.6 [22.4, 29.3]
NFHS 5	Other	19.3 [18.5, 20.1]	21.4 [20.4, 22.4]
	Other Backward Class	21.6 [20.8, 22.5]	23.3 [22.3, 24.4]
	Scheduled Caste	22.6 [21.7, 23.5]	24.6 [22.3, 27.1]
NFHS 5	Scheduled Tribe	23.5 [22.4, 24.6]	23.5 [20.2, 27.3]
C) Wasting			
**NFHS**	**Caste**	**Hindu** **Prevalence [95%CI]**	**Muslim** **Prevalence [95%CI]**
NFHS 1—5	Other	11.4 [11, 11.9]	11.6 [11.1, 12.2]
	Other Backward Class	12.4 [11.8, 12.9]	12.6 [12, 13.2]
	Scheduled Caste	14.1 [13.5, 14.9]	13 [11.4, 14.8]
	Scheduled Tribe	12.6 [12.1, 13.2]	12.2 [11.1, 13.4]
**NFHS**	**Caste**	**Hindu** **Prevalence [95%CI]**	**Muslim** **Prevalence [95%CI]**
NFHS 1	Other	16.2 [14, 18.8]	16.8 [14.4, 19.7]
	Other Backward Class		
	Scheduled Caste	17.4 [14.7, 20.5]	19.8 [11.1, 35.3]
	Scheduled Tribe	20.3 [16.9, 24.3]	3.6 [0.1, 104.2]
NFHS 2	Other	7.2 [5.9, 8.7]	7.5 [6.1, 9.2]
	Other Backward Class	8 [6.6, 9.7]	8.1 [6.3, 10.4]
	Scheduled Caste	7.8 [6.4, 9.6]	6.5 [3.3, 12.5]
	Scheduled Tribe	9.2 [7.4, 11.4]	9.2 [4.1, 20.8]
NFHS 3	Other	11 [9.7, 12.5]	11.2 [9.8, 12.9]
	Other Backward Class	11.4 [10.1, 12.9]	11.2 [9.7, 12.9]
	Scheduled Caste	12.3 [10.8, 13.9]	13.5 [9.4, 19.2]
	Scheduled Tribe	13.7 [11.9, 15.7]	14.3 [7.4, 27.5]
NFHS 4	Other	14.7 [14, 15.5]	14.3 [13.5, 15.2]
	Other Backward Class	15.4 [14.7, 16.1]	15.1 [14.3, 16]
	Scheduled Caste	15.4 [14.7, 16.2]	13.5 [11.7, 15.6]
	Scheduled Tribe	17.6 [16.6, 18.6]	16.9 [14.2, 20.1]
NFHS 5	Other	14 [13.3, 14.8]	14.5 [13.6, 15.4]
	Other Backward Class	15.5 [14.8, 16.3]	16.4 [15.5, 17.4]
NFHS 5	Scheduled Caste	16 [15.2, 16.8]	16.9 [14.8, 19.3]
	Scheduled Tribe	17.3 [16.3, 18.4]	16.7 [13.7, 20.4]

In the case of tribes, Hindu STs had a higher prevalence of stunting [40.6% (95%CI: 39.4, 41.9)] compared to Muslim STs [39.7% (95%CI: 37.2, 42.4)], and comparable prevalence of underweight (Table 3B and C). While data in religion-tribe strata was sparse resulting in overlapping confidence intervals, for wasting, Muslim STs had better or comparable prevalence to Hindu STs (Fig. 1).

Relative to Hindu-Other caste children, risk ratios of stunting in Muslim Other castes, Muslim OBCs, Muslim identifying as SCs, and Muslim STs were 1.16 (95%CI: 1.13, 1.18), 1.17(95% CI: 1.14,1.19), 1.17(95% CI: 1.10,1.24) and 1.14 (95% CI: 1.04,1.24); and Hindu OBCs, Hindu SCs and Hindu STs were 1.11(95% CI: 1.09,1.13), 1.17(95% CI: 1.15,1.19), and 1.15 (95% CI: 1.12, 1.17), respectively (Table S3 (A)) (Fig. 1). Estimated risk ratios for underweight and wasting are presented in Table S3B.

Across the three outcomes, the directions of the estimated referent effects for religion and caste and tribal identities were opposite to the interaction estimates for religion and caste/tribe, indicating sub-additive or antagonistic interactions [60]. For stunting, the ratio of RRs or estimated relative risks for religion and OBC compared to Other (forward) castes were 0.91 (95% CI: 0.88, 0.94), SCs/Dalits and STs was 0.86 (95% CI: 0.81,0.92), and 0.86 (95% CI: 0.78, 0.94) (Table 4) (Table S3). This negative interaction suggests that the estimated joint effect of caste or tribal identity and Muslim identity was lower than the product of the estimated effects of caste/tribal identity and Muslim identity alone (Table S3). Thus, the risk ratio of stunting associated with Muslim and minority caste or tribe together or synergistically, was lower than the product of estimated risk ratios of stunting associated with being Muslim alone, or being a (Non-Muslim) minority caste or tribe alone.

**Table 4:** Joint disparity, referent religion disparity, referent caste, and referent tribal identity disparity for predicted prevalence of child growth outcomes in adjusted models. These estimates are adjusted for household wealth, mother's education, mother’s height (for stunting and wasting), mother’s weight (for underweight and wasting), child’s age, child's sex, urbanicity. We also use state and district fixed effects, and included each NFHS survey wave as a fixed effect to control for all state invariant factors that may vary over time

	**Other Backward Class**			
**Variable**	**Joint disparity**	**Referent Muslim disparity**	**Referent social group disparity**	**Excess intersectional disparity**
% [95% CI]	µ11 − µ00	µ10 − µ00	µ01 − µ00	µ11 − µ10 − µ01 + µ00
Stunting	0.17 [0.14,0.19]	0.16 [0.13,0.18]	0.11 [0.09,0.13]	-0.10 [-0.08,-0.12]
Underweight	0.16 [0.13,0.19]	0.10 [0.08,0.13]	0.12 [0.1,0.13]	-0.06 [-0.05,-0.07]
Wasting	0.1 [0.07,0.14]	0.01 [-0.02,0.04]	0.08 [0.06,0.11]	0.01 [0.03,-0.01]
	**Schedule Tribe**			
**Variable**	**Joint disparity**	**Referent Muslim disparity**	**Referent social group disparity**	**Excess intersectional disparity**
% [95% CI]	µ11 − µ00	µ10 − µ00	µ01 − µ00	µ11 − µ10 − µ01 + µ00
Stunting	0.14 [0.04,0.24]	0.16 [0.13,0.18]	0.15 [0.12,0.17]	-0.17 [-0.25,-0.15]
Underweight	0.16 [0.06,0.28]	0.1 [0.08,0.13]	0.23 [0.2,0.25]	-0.17 [-0.22,-0.06]
Wasting	0.14 [0.01,0.29]	0.01 [-0.02,0.04]	0.23 [0.2,0.27]	-0.1 [-0.17,-0.02]
	**Schedule Class**			
**Variable**	**Joint disparity**	**Referent Muslim disparity**	**Referent social group disparity**	**Excess intersectional disparity**
% [95% CI]	µ11 − µ00	µ10 − µ00	µ01 − µ00	µ11 − µ10 − µ01 + µ00
Stunting	0.17 [0.1,0.24]	0.16 [0.13,0.18]	0.17 [0.15,0.19]	-0.16 [-0.18,-0.13]
Underweight	0.2 [0.13,0.28]	0.1 [0.08,0.13]	0.17 [0.15,0.2]	-0.07 [-0.05,0.02]
Wasting	0.06 [-0.03,0.16]	0.01 [-0.02,0.04]	0.11 [0.08,0.13]	-0.06 [-0.16,-0.07]

Negative interactions were also observed on the additive scale. The estimated RERIs relative to Hindu Other Castes were -0.10 (95% CI: -0.13, -0.07) for Muslim OBCs, -0.16 (95% CI: -0.23, -0.09) for Muslim Dalits, and -0.17 (95% CI: -0.27, -0.06) for Muslim STs (Table S3), again indicating that the estimated joint effect on the additive scale of minority caste/tribal identity and being Muslim was lower than the sum of the estimated individual effects of minority caste/tribal identity or religion alone.

Similar directions and effect sizes of interaction effects were estimated for underweight (Table 4) (Table S3(B)). For wasting, effect sizes were in the same range, but not statistically significant for OBCs and SCs (Table 4) (Table S3(C)).

### Decomposing joint disparities into component and excess intersectional disparities

The joint disparity is the total difference in the prevalence of outcomes between doubly marginalized (Muslims and OBC/SC/ST) compared to non-marginalized children (Hindu forward castes) [35, 37]. The referent disparities refer to the disparities attributed to disadvantages of minority religion only, or deprived caste or deprived tribe only. The excess intersectional disparity is the amount of the total difference that is attributable to being both a religious and a cast or tribe minority [35].

Again, taking stunting as an example, the joint disparity for Muslim OBC, SC and ST children, each relative to Hindu Other castes children was 0.17(95% CI: 0.14, 0.19), 0.17 (95% CI: 0.10, 0.24), and 0.14 (95% CI: 0.08, 0.20) on the RR scale (Table 4). The referent religion (Muslim) disparity was 0.16 (95% CI: 0.13,0.18) and the referent OBC, SC and ST disparities were 0.11 (95% CI: 0.09,0.13), 0.17 (95% CI: 0.15,0.19) and 0.15 (95% CI: 0.12,0.17) respectively (Table 4). The estimated excess intersectional disparity for Muslim OBC, SC and ST were -0.10 (95% CI: -0.09, 0.13), -0.16 (95%CI: -0.23, -0.09), and -0.17(95% CI: -0.27, -0.06) (Table 4). Estimated referent disparities associated with prevalence of underweight and wasting are also presented in Table 4.

### Exploring heterogeneities by strata of household, maternal and child characteristics

We examined heterogeneities for intersectional strata defined by religion-social group and each of the following variables which have been independently associated with disparities in anthropometric outcomes- household wealth, mother’s education, and child’s sex and child’s age (Figure S2). For every religion-caste and religion-tribe strata, poorer households, and children of mothers with lower education levels have a higher burden of stunting, underweight and wasting, relative to richer households, and with mothers of higher education. We identified no statistically different patterns in these heterogeneities for different Muslim-caste and Muslim-tribe subgroups (Figure S2). Among child characteristics, while boys had a higher prevalence of stunting, underweight and wasting, confidence intervals in estimated prevalence were overlapping (Figure S2). Muslim SCs was the only exception, where girls had higher or comparable prevalence to that of boys for all three outcomes (Figure S2). Older children have a higher burden of the outcomes compared to younger children (Figure S2).

### National intersectional disparities in child growth by strata of religion-caste and religion-tribe over time

Across outcomes, between caste or tribal identity differentials by religion and were more precisely estimated in the two most recent survey waves (Fig. 3). Between NFHS 1 (1992–93) to NFHS-5 (2019–21), the Hindu advantage in predicted prevalence of stunting in percentage points increased from 1.6 to 3.7 for Other (forward) castes, reduced from 3.4 to 2.3 percentage points for OBCs (Table 3). For SCs, Muslims had an advantage of 1.5 percentage points in NFHS 1, which switched to a Hindu one of 1.8 percentage points in NFHS 5 (Table 3). For STs, the Muslim advantage reduced from 2.6 to 1.3 percentage points (Table 3).

**Fig. 2:**
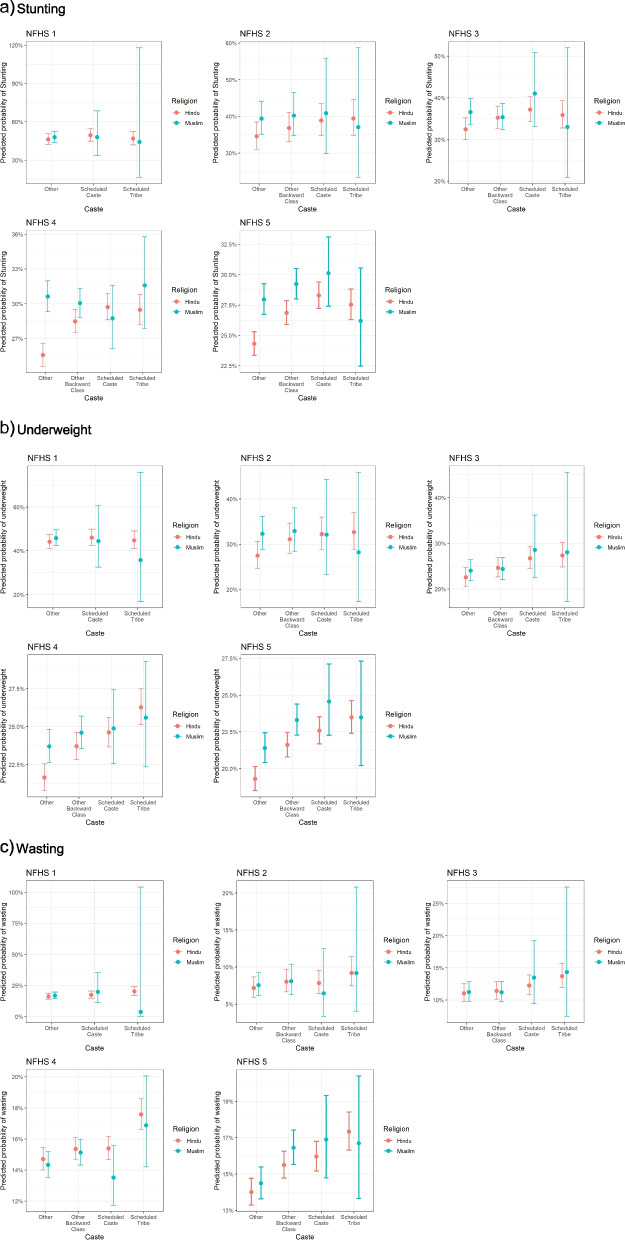
Predicted prevalence of child growth outcomes within strata of religion -caste and religion-tribe stratified by each wave of the National Family Health Survey (NFHS 1: 1992–93, NFHS 2: 1998–99, NFHS 3: 2005–06, NFHS 4: 2015–16, NFHS 5: 2019–21): a) stunting, b) underweight, c)wasting. These estimates are adjusted for household wealth, mother's education, mother’s height (for stunting and wasting), mother’s weight (for underweight and wasting), child’s age, child's sex, urbanicity. We also use state and district fixed effects

The Hindu advantage in the predicted prevalence of underweight in percentage points increased from 1.7 to 2.1 for Other Castes, reversed from a Muslim to a Hindu advantage for SCs from 1.5 to 2 percentage points respectively, reduced from a Muslim advantage of 9.1 to comparable Hindu-Muslim prevalence in NFHS 5(Table 3) (Fig. 2). For OBCs between NFHS 2 and 5, the Hindu advantage reduced marginally from 1.8 to 1.7 percentage points (Table 3). For wasting, Hindu-Muslim differentials across social groups remained more consistent between NFHS 1 and 5 (Table 3). In the last two waves, highest Hindu advantages were observed for Other (forward) castes and OBCs (Fig. 2). For STs similar time trends were observed, but with a Muslim advantage (Fig. 2). However, given the small sample size of Muslim STs, our estimated CIs were wide (Fig. 2).

### Intersectional disparities in child growth by social strata of religion-caste and religion-tribes in states and union territories

**Fig. 3:**
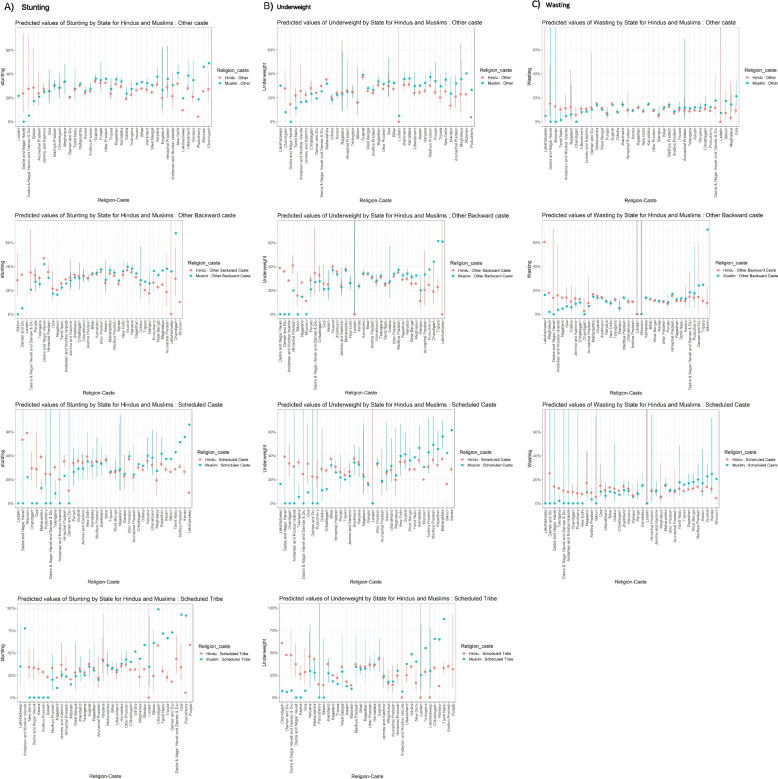
Predicted child growth outcomes in strata of religion and social group by States and Union Territories (ordered in increasing order of Hindu-Muslim differential): **a**) stunting, **b**) underweight, **c**)wasting. These estimates are adjusted for household wealth, mother's education, mother’s height (for stunting and wasting), mother’s weight (for underweight and wasting), child’s age, child's sex, urbanicity. We also use state and district fixed effects, and included each NFHS survey wave as a fixed effect to control for all state invariant factors that may vary over time

States reflected national trends to varying extents. For stunting, among forward castes, Hindu children generally had a lower prevalence than Muslims across caste and tribal identities, with high differentials in Delhi, Bihar, Uttar Pradesh, West Bengal and Assam, and lower differentials in Madhya Pradesh, Chandigarh, Kerala, among others (Fig. 3). However, there were exceptions. For example, In Maharashtra, Hindu other (forward) caste children had a higher prevalence of underweight (Fig. 3). Muslim Other (forward) castes had a lower prevalence of outcomes than their Hindu counterparts in Jammu and Kashmir. For OBCs, the Hindu-Muslim differentials had lower effect sizes and were less precisely estimated, but were also highest in Gujarat, Haryana, and Rajasthan (Fig. 3). In Telangana, Punjab, and Jammu and Kashmir, Hindu OBCs had a higher prevalence of stunting than Muslim OBCs, while Hindu and Muslim OBCs were comparable in Uttar Pradesh(Fig. 3). In Rajasthan, and Haryana, Muslim OBCs had a higher prevalence of both stunting and underweight compared to Hindu OBCs(Fig. 3). For SCs and STs, state level Hindu-Muslim differential estimates were least precisely estimated, possibly due to the smaller sub-sample of Muslim SCs and STs. Since confidence intervals of both groups overlap, we cannot report statistically significant trends for these groups from any states (Fig. 3). However, Muslims SCs generally tended to have higher prevalence than Hindu SCs, and more so in Tamil Nadu and Rajasthan (Fig. 3). For STs in Jammu and Kashmir and Jharkhand, the trend reversed with higher prevalence in Hindu STs compared to Muslim STs ( Fig. 3).

Across states and across outcomes, Muslim Other (forward) castes had comparable prevalence to Hindu deprived castes, including Hindu OBCs, and in some cases even Hindu SCs (the most disadvantaged caste). For example, for stunting, in Uttar Pradesh while Muslim other castes had an estimated prevalence of 36% (95% CI: 35.0,38.0), Hindu OBCs and Hindu SCs had a prevalence of 37% (95% CI: 35.0, 38.0) (Fig. 3). In West Bengal, Muslim Other (forward) castes had an estimated prevalence of 30% (95% CI: 29.00, 31.00), compared to Hindu OBCs’ estimated 21% (95% CI: 20.00, 22.00) (Fig. 3).

### Sensitivity analysis

Since some covariates could be in the pathway between caste/tribe and religion identity and child’s anthropometry, we estimated unadjusted interactions for religion and caste on child anthropometry (Table S2). The main referent effects for religion and social groups were attenuated after the inclusion of covariates, indicating the covariates explained some, but not all the intersectional religion-social group disparities (Table S2).To understand how far our intersectional estimates could be driven by changes in the composition of OBCs, we restricted the sample to NFHS 4 and 5, when the distribution of sampled children across castes/tribes were largely consistent (Figure S1). We found that our estimates of additive and multiplicative interaction as well as joint, referent and intersectional disparities in this restricted sample were consistent with the larger sample. For example, for stunting, the multiplicative interaction estimates for OBC and Muslim identity were 0.92 (95% CI: 0.80, 0.95) compared to 0.91 (95% CI: 0.88, 0.94). Additionally, while sample sizes for different strata were small at the level of states and do not allow us to make very conclusive assessments, we found state level trends may be different from national intersectional trends for all strata (including but not restricted to OBCs) (Fig. 3).

## Discussion

Based on the framework of intersectionality and using data from nationally representative surveys spread over 30 years in India, we reported how joint and simultaneous social privileges accorded by religion and caste, and religion and tribe as intersecting social strata, were associated with disparities in child growth outcomes for Hindu and Muslim children under 5 years of age. For stunting and underweight, while Hindu children had an advantage over Muslim children who shared their caste or tribal group affiliations, the magnitude of this advantage was the highest for the most privileged social group, the forward (other) castes. Although data was sparse for strata at the intersections of religion-tribes, Muslim tribal children appeared to have better or comparable outcomes to Hindu tribal children. Finally, for wasting, estimated prevalence for religion-social group strata were directionally similar, but less conclusive. These findings support religion and caste, and religion and tribe, as intersectional determinants of structural inequities, that warrant joint consideration in monitoring and policies to effectively target disparities in child growth in India.

A few specific intersectional findings are of note, which together underscore Bowleg et al.’s exposition of how intersecting social positions inform or “constitute” each other in their influence on health disparities [2, 26]. Thus, isolating how relative privileges or disadvantages associated with religion or social group alone are associated with health disparities obfuscates the true picture of how social position determines health disparities in India [2]. First, Muslim forward caste children were not only disadvantaged relative to Hindu forward caste children. They also lagged Hindu children from disadvantaged castes (SCs and OBCs) in their predicted prevalence of stunting (Fig. 1). Additionally, their predicated prevalence of underweight was comparable to Hindu children from deprived castes (Fig. 1). This indicates that for Muslim forward caste children, structural disadvantages associated with their Muslim identity may be taking precedence over any advantages accorded by their privileged caste status [14]. This is supported by lagging socioeconomic indicators of Muslims across different caste and tribal groups, in literacy, educational outcomes, household wealth and ownership of assets [22]. All Muslims also face other structural hardships including ghettoization, communal violence, and interpersonal discrimination, associated with their social identity as Muslims [58, 59]. For example, tenants who have Muslim names have a hard time finding rental accommodations in many metropolitan cities in India [61]. Ethnographic research from Indian villages has explored how spatial organization of villages reflects the complex intersecting social hierarchies of religion and caste for Muslims [5]. While forward caste Hindu homes are situated “on one side of the road”, all Muslims and lower caste Hindus are on the other side. On their lane, Muslim forward caste pockets are spatially distinct from both Hindu and Muslim backward caste hamlets [5]. However, Muslim forward caste communities still reside “outside the main village”, closer to the homes of the least privileged Hindu castes, often in areas with poor or absent drainage, fractured roads, with no streetlights [5]. Moreover, aspects of untouchability, a social practice otherwise associated with the caste system, sometimes presents in interpersonal discrimination experienced by all Muslims [20]. For example, Hindu forward caste households may not eat in the homes of any Muslims, “irrespective of their caste” [5].

Second, Hindu and Muslim children from disadvantaged castes and tribes had more comparable prevalence of stunting, underweight and wasting, compared to Hindu and Muslim children from forward castes (Fig. 1). This could indicate that structural deprivations associated with disadvantaged caste or tribe, override relative social advantages of Hindu identity. While Muslim children from disadvantaged social castes who face the dual social disadvantages of religion and social group strata, still lagged Hindu deprived castes, the magnitude of this differential was less than that for Muslim forward caste children (Fig. 1). Ethnographic research from different parts of the country has documented how Muslim backward caste communities are subject to discrimination within the Muslim society. In West Bengal, Muslim forward castes do not eat with Muslim backward castes [20]. Studies in Tamil Nadu and Kerala have outlined how Muslims of forward caste do not marry Muslims of backward castes, rationalizing endogamy with a belief about proximity of advantaged castes to the Prophet [20]. Similarly, Muslim backward communities face similar relative economic disparities as Hindu deprived caste communities, in ownership of land, property and disproportionate representation in occupations like manual labor, sanitary work, and unorganized farm labor [4]. As such, Muslim backward caste communities have been organizing for political representation and demanding constitutional safeguards guaranteed to Hindu deprived caste communities [14]. The relative social and economic position of Muslim backward caste communities also remains behind other Muslims [4, 22].

Third, our findings also underline the distinctly advantageous outcomes of the dually privileged Hindu Other (forward) caste children, which has also increased over the period of analysis from 1992–92 to 2019–21, in some cases (Fig. 2). These social advantages are reflected in the higher socioeconomic, employment, educational indicators of Hindu forward caste communities [28, 45]. Other structural advantages include their better political participation and representation in the electorate, judiciary, administrative positions, ownership of land, and access to health [45]. Finally, given the close association of household wealth and child growth [11], the higher intergenerational economic mobility in these dually privileged communities relative to other social group-religion strata, could explain the increase in their relative social advantage in the prevalence of stunting and underweight over the period of analysis (Fig. 2) [11].

Fourth, while intersectional trends by strata of tribe and religion varied from health disparities for religion-caste strata, they again reflected the need to jointly consider these identities when planning tribal health policies. Hindu tribes had higher prevalence of stunting, underweight and wasting, compared to Muslim tribes (Fig. 1). However, among all Hindu children, tribal children did much worse than children from other social groups, for all three outcomes (Fig. 1). In comparison, growth outcomes of Muslim tribal children were comparable to those of Muslim children from other social groups (Fig. 1). For stunting, Muslim tribes were even comparable to Muslim forward caste children (Fig. 1). Somewhat parallel to intersectional trends for religion-caste, we again see that social disadvantages associated with tribal identity seemed to take precedence over social advantages from Hindu identity, for Hindu tribal children (Fig. 1). For Muslim tribal children, Muslim social identity seemed to accord some social advantages to their health outcomes (Fig. 1). Tribal communities in India live in rural and remote areas, and have historically had the poorest literacy and high poverty levels, despite affirmative action policies, and various longstanding and intermittent tribal welfare programs in education, health and nutritional support for tribal women and children, in different states [9, 62]. Additionally, while protected tribes are identified under the single constitutional category of Schedule Tribes(STs), there is vast heterogeneity in their composition, internal social hierarchies, and food habits, all or any of which could be playing a role in these intersectional trends [9]. Finally, Muslim STs are somewhat geographically restricted to three major regions of the country- Jammu and Kashmir which is a union territory that has a majority Muslim population in the north, Maharashtra in the west and Lakshwadeep island [62]. The relative advantages of Muslim STs could be reflective of state or regional effects. At the same time, historically Muslim STs have been the beneficiaries of affirmative government policies and interventions, unlike Muslims identifying as SCs [63]. Thus, the comparatively improved outcomes of Muslim STs could suggest that with similar constitutional safeguards, outcomes of Muslim SCs could also see improvements [14]. An important limitation for these interpretations is that Muslim tribes as an intersectional social stratum, had the lowest sample size (Table 1), which makes it difficult to draw conclusive interpretations on this particular religion-social group strata.

Somewhat contradictory to other quantitative applications of intersectional decomposition analysis following Jackson et al.’s approach [35, 37], our estimates of interaction effects of religion and social group, also known as the excess intersectional disparities associated with these intersecting strata, were positive, while the estimated main effects of religion and all social groups were negative (Table 4, Table S3) [35]. In epidemiological terms, this suggests sub-additive or antagonistic interactions where the direction of interaction effects is opposite to that of main effects [60]. However, the doubly marginalized subgroups in our analysis, Muslims of deprived castes (SCs and OBCs) were disadvantaged compared to Hindus of the same caste, for both underweight and wasting (Fig. 1). However, in the case of tribes, Hindu STs were disadvantaged compared to Muslim STs (Fig. 1). We also found that disparities associated with Muslim identity alone (referent disparities or the main effects for Muslim identity) were higher in magnitude to disparities associated with OBC identity alone, and comparable to that of SC and ST identity alone (referent disparities or the main effects for each social group) (Table 4, Table S3).

Together, this intersectional decomposition analysis suggests that Muslims have an overall social disadvantage in child anthropometric outcomes, which is comparable in magnitude to the deprivations associated with backward caste and tribal identities alone [57]. This could mean that disadvantages associated with Muslim identity may have variable impacts on child growth across different backward caste and tribal groups [37]. Muslim identity appears to be especially disadvantageous in corroding the benefits of forward caste identity for Muslim forward caste children, as indicated by their high disparities relative to Hindu forward caste children (Table 3, Fig. 1). As discussed earlier, ethnographic evidence from the region suggests that backward caste communities of both Hindu and Muslim religions face similar social oppressions, which may be reflected in their more comparable outcomes [16]. Another way to understand this could the apparently protective role of Muslim identity for deprived castes, which is reflected in the lower disparities of Muslim backward caste children, compared to their Hindu counterparts (Fig. 1). This could suggest that Hindu backward caste children face more caste based discrimination within their religion, compared to the experiences of Muslim backward caste children, within Muslim society [64]. This could be supported by the ideas of power and purity rooted in the Hindu caste system, which gave rise to social ills like untouchability toward deprived castes [42]. While social stratification in Muslim society in India also reflects some of these concepts, especially in communities that have historically converted from Hinduism, it is also based on other ideas like beliefs of relative proximity of different communities to the Prophet [20]. At the same time, the poor conditions of Muslim children from deprived social groups needs to be underscored. Unlike Hindu deprived caste communities who have benefited from constitutionally guaranteed affirmative action policies, protections granted to Muslim OBCs varies by states and has not been consistent since independence [14]. Moreover, communities identifying as Muslim SCs are not legally recognized as a protected category, or guaranteed affirmative action policies [63].

Motivated by the roots of intersectionality in the Black Feminist movement, we also examined how other dimensions of social stratification, particularly maternal education, influenced our estimated disparities at the intersection of religion and social group identities (Figure S2). For every religion-caste and religion-tribe strata, we found children born to mothers with lower education levels had a higher burden of these outcomes, relative to children who had more educated mothers (Figure S2). However, this meant that children who were deprived in religion and caste or religion and tribe, who were already behind children from more advantaged strata, did especially poorly, when they were born to mothers with lower education (Figure S2). This suggests that policies to improve educational outcomes of women, may improve children’s health disparities for all intersecting religion and social group strata, but may be especially beneficial for children at the marginalization of these dual strata. Social hierarchies associated with caste, tribe and religious identity are inherently patriarchal and accord a lower social status to women of minority social and religious identity, compared to men [41]. As an example, this has resulted in more women from disadvantaged castes being forced into “polluting” professions like sanitary work, compared to men of the same caste [39]. Some of these deprivations also manifest in how women from deprived castes and minority religions have poorer educational outcomes, face increased exposure to violence, and a higher risk of maternal mortality, compared to Hindu women from more advantaged castes [41, 65, 66]. At the same time, studies in multiple low-income country contexts have shown improving literacy and educational outcomes of women have beneficial intergenerational health outcomes, including better health seeking behavior, higher future institutional births, improved menstrual hygiene, better immunization rates, lower short and long term morbidity for women and children [67, 68]. Across strata of religion-caste and religion-tribe, we also found that children from poorer households had lower prevalence of growth outcomes compared to children from richer households (Figure S2). Thus, while poverty alleviation programs could benefit children across strata of religion-caste and religion-tribe, these efforts could especially help boost outcomes for children at the intersections of deprived castes, tribes, and religion [69].

Among child characteristics, for all subgroups except Muslim-SCs, male children had a higher prevalence of stunting, underweight and wasting, although confidence intervals were overlapping with estimates for female children (Figure S2). In most countries, prevalence of early childhood undernutrition has been found to be higher in male children, similar to other outcomes in this age group like infant and child mortality [70]. While social and contextual factors have been somewhat distinguished from biological ones in efforts to understand these trends, the need for more detailed investigation into gender related pathways, separately from biological and immunological mechanisms, has been underscored [70]. So far, these male–female differences have been attributed to multiple hypotheses, including biological disadvantages of male fetuses, and selection patterns in historical son preferences, that allowed female children to be born to caregivers likely to provide them nurturing care and support [70].

We found more conclusive evidence of religion and caste interactions for stunting and underweight, compared to wasting. While stunting is indicative of long-term nutritional deprivation with lasting consequences for growth potential and cognitive child development, wasting is reflective of short term disruptions in nutrition and/or acute infections like diarrhea [47]. Underweight is a comprehensive indicator capturing both these indicators [71]. Thus, our stronger interaction estimates for stunting and underweight could indicate that relative intersectional social advantages accrued through multiple generations, influence longer term child growth deprivations more than short term nutritional interruptions [32]. This would align with sociocultural models of child development and the ecological systems theory, which posit that relative privileges and exploitations determined by social positing are accrued over time and generations [32, 72].

Trends in our intersectional estimates during our 30-year period of analysis, show that Hindu-Muslim disparities across social groups become more precise and larger in effect size (Fig. 2). For stunting, between NFHS 1 and 5, Hindu advantages have more than doubled for other (forward) castes, reduced marginally by 1 percentage point for OBCs (between NFHS 2 and 5). Moreover, between caste/tribal group differences in anthropometric outcomes between Hindu and Muslim children have seen the most changes in stunting, and largely to the disadvantage of doubly marginalized subgroups (Fig. 2). As an indicator of multiple disadvantages accrued over time, stunting may impact children’s long term growth and cognitive development potential [73]. These trends could partially be driven by larger sample sizes in the last two surveys in 2015–16 and 2019–21. Other Backward Classes (OBC)s are the only social group for which the Hindu advantage seems to have reduced over the period of analysis (Fig. 2). The estimated Muslim advantages over Hindus for Schedule Castes (SCs) in NFHS 1 (1992–93) reversed in favor of Hindus by NFHS 5 (2019–2021) (Fig. 2). The advantage Muslim tribal children had over Hindu children, reduced in magnitude in the case of tribes (STs) (Fig. 2). Finally in the case of wasting, we found less pronounced evidence of intersectional advantages and by strata of religion and social group especially in the early survey rounds (Table 3, Fig. 2).

While magnitudes of differentials varied in different states, they reflected national trends with some variations. Muslim advantages in Other, OBCs and STs were noted in Jammu and Kashmir, and some north-eastern states. In Maharashtra and Chhattisgarh, there was higher prevalence of stunting among Muslim Other castes, but higher prevalence of underweight among Hindu other castes (Fig. 3). This may indicate the need for localized policies to better target inequities determined by religion and social group differentials. The recognition of OBC status varies by states [14]. In some states like Uttar Pradesh, where Muslims have historically been granted OBC status, the outcomes of Muslim and Hindu OBCs are relatively similar, although collectively poor (Fig. 3). However, this is not true of all states. In Haryana and Rajasthan, Muslim OBCs continue to far poorer than Hindu OBCs (Fig. 3).

We did not quantitatively examine mechanisms of estimated intersectional health disparities. However, a broad scholarship has linked religious affiliation, religiosity, and religious identity with health behaviors and outcomes [29]. Self-identification as a religious minority has been associated with adverse child health [74]. Weller and colleagues posit that religious disadvantage occurs when some groups have “privileged arrangements” with institutional power and policy in multireligious societies [75]. The “complex religion” theory posits religion as a key determinant of the “racialization process”, that creates and maintains other social hierarches [65]. In India and South Asia, caste and religion have interacted and mutually reinforced deep rooted hierarchies that determine access to wealth, education, power and intergenerational mobility for generations [23]. These intersections also influence other structural factors like exposure to community violence, support from social networks and social cohesion, among others [24]. Importantly, the sociocultural environment also interacts with the household environment in influencing caregiver behaviors [31]. Finally, religion, caste and tribal identities also collectively inform habits like diet, drinking and smoking, as well as sanitation practices [76]. This intersectionality also determines gender norms that influence women’s access to education which influences rates of immunization patterns and nutritional support for children [68].

Our analysis has several policy implications. The heterogeneities in patterns of child growth by religion-caste and religion-tribe strata suggests the need to incorporate this intersectionality in the measurement of administrative and health related data. Currently, Muslims are treated as a monolith in all routine monitoring of health data, where their indicators are aggregated across social groups [14, 15]. Such an approach may obfuscate important subgroups, which may require potentially different policy approaches. For example, given the poor outcomes of doubly disadvantaged Muslim children of backward castes, they may require explicit focus in policies like the national nutrition mission that are aimed to reduce disparities in children’s undernutrition. Similarly, given their relatively poor outcomes, Hindu tribes may require special attention compared to tribes who are Muslim. Moreover, Muslim deprived caste communities are not legally recognized as SC or accorded constitutionally mandated affirmative action policies granted to Hindu SCs, although Muslim communities recognized as OBCs are granted some of these protections [63]. Given the poor outcomes of Muslim SCs and Muslim OBCs compared to Hindu SCs and Hindu OBCs in our analysis, these communities may require added support. There has been a longstanding demand from Muslim backward caste communities to be granted similar protections as SCs [16]. Finally, OBC status varies by state with some communities who are recognized as OBC in specific states, may not be granted this legal protection in other states. As stated above, Uttar Pradesh, where Muslims have historically been granted OBC status, the outcomes of Muslim and Hindu OBCs are similar, although poor (Fig. 3). In states like West Bengal, where the share of Muslim OBCs has seen recent depletions, their outcomes remain poorer than Hindu OBCs (Fig. 3). Thus, our findings may lend some support the benefits of being recognized as OBC for Muslims. Finally, given the poor outcomes of Muslim forward caste children relative to Hindu forward caste children, and in some cases, even Hindu deprived caste children, all Muslim may need targeted policy action, with even special focus on Muslim children of deprived castes [22, 63]. This may mean more targeted nutrition sensitive and nutrition specific policies, as well as access to constitutional protections towards improving the community’s literacy and socioeconomic conditions [14, 15]. While our analysis was focused on Muslim children, recommendations to consider religion-caste and religion-tribe as intersectional social strata in policies to monitor and target disparities, likely also apply for Christians, due to the similar historical antecedents of social stratification in both religions in the Indian subcontinent [77]. Similar to Muslims, Christian deprived castes are also not granted SC status or its associated constitutional protections [15, 77]. Thus, our findings call for a historically informed, and contextually aware revision in public health monitoring and policies, that incorporates intersections of lived social group experiences across religions to better measure and address child health disparities. Importantly, we also found relative increase in women’s education was associated with improved child growth across strata of religion-caste and religion tribe (Figure S2). Women from minority castes and minority religions face dual marginalization in their poorer literacy, poor maternal outcomes, and higher exposure to violence within and outside the household [41]. At the same time, maternal education has been associated with improved childhood immunization, better nutritional support and improved access to health for children [78]. Thus, the interaction patterns of strata formed by maternal education and minority religious and social identity in our analysis highlight that improving women’s education across these social strata could improve women’s social outcomes and target child health disparities associated with this multiple marginalization.

Our interaction estimates should be considered in the context of changes in the composition of social groups through the thirty-year period of our analysis. First, OBCs were officially recognized as a disadvantaged social group only in 1990 [13]. Between NFHS 1 and 2, disparities between Hindu-Muslim Other(forward) caste and OBCs became more pronounced, but confidence intervals remained overlapping (Fig. 2). Furthermore, for SCs, a possible Hindu disadvantage in NFHS 1 reversed to a possible Muslim 1 in NFHS 2 (Fig. 2). Moreover, unlike SCs and STs, the OBC category is a transient one, with communities being included over time and across states [13]. And indeed, proportion of sampled OBCs increased over time among Muslims (Figure S1). Thus, trends over time should be interpreted with caution. However, our primary analysis was largely restricted to national trends. We also conducted stratified analysis for each NFHS wave, and our analysis was robust to restricting the sample to the last two waves when number of sampled OBCs among Hindus and Muslims was more consistent (Figure S1). Moreover, if the most disadvantaged communities moved from Other castes to OBCs uniformly for both Hindu and Muslim children, these changes are unlikely to have differentially altered estimates of Hindu-Muslim child health disparities across social groups.

Our analysis has several limitations. Measurement of caste, tribal and religious identity may be subject to measurement errors since NFHS only relies on self-reported OBC, SC or ST status [46]. Additionally SC status is not constitutionally granted to Muslims and Muslim SCs are not a legally recognized social group [79]. Some deprived caste Muslims have been granted OBC status in some states but not in others, making OBC Muslims a more heterogenous category compared to OBC Hindus. Since the OBC category also varies over time, trends over time should be interpreted with caution. In reporting estimates by population subgroups, our descriptive work is an important first step in quantitatively assessing our intersectionality hypothesis. While we discuss theoretical positions of power and privilege to explain our estimated intersectional trends, we do not quantitatively estimate mechanisms such as religious or caste-based discrimination. Future work should attempt to measure how religious or social group based discrimination may be causing health inequities by these intersectional experiences. Additionally, considering the historical roots of intersectionality in gender as a predictor of access to power and privilege, and deep rooted patriarchal practices associated with both religion and caste, future scholarship should study intersections of religion-social group subgroups with variables capturing women’s social status and gender roles in the household and community [41]. These could include measures like women’s literacy and education, women’s occupation, women’s role in household decision making, as well as women’s exposure to domestic violence [41]. Moreover, our estimated intersectional health disparities should be validated with other surveys, with different sampling designs or administrative data. Finally, given that recent studies have shown depletions in the ‘Muslim advantage’ in child mortality [80], examining other child development outcomes by intersectional religious and social group strata could also be helpful.

Our study also has several strengths. We used the intersectionality framework in a non-western setting, based on a theoretically driven, contextually relevant hypothesis. We also included minority religious identity as a dimension of intersectional inequities, in response to calls to incorporate newer dimensions of social identity to augment global understanding of intersectional health inequities [28]. We studied patterns of intersectionality nationally, as well as by states and reported trends over a 30-year period, with the objective of identifying vulnerable subgroups who would benefit from targeted approaches. We examined estimates of interaction on both short and long term indicators of child growth, and estimated their state variations, thus examining contextual variation in intersectionality. Finally, we reported conservative estimates of interaction after “controlling” for household socioeconomic status, maternal education, and child level characteristics. Since many of these variables could be potential mediators in how these intersectional lived experiences influence child growth outcomes, our estimates of health disparities are possibly underestimated.

## Conclusion

Religion-caste and religion-tribe are important social strata that simultaneously influence child health inequities. Epidemiological analysis to measure and intervene on health disparities from the region should embrace a historically informed and theoretically driven approach that incorporates the joint lived experiences of relative social privilege and disadvantage from religion-caste and religion-tribe identities.

## Supplementary Information

**Additional file 1:Table S1.** List of caste and tribal social groups that have been estimated in their intersections with two religious identities (Hindus and Muslims) as social strata jointly informing intersectional child health disparities. **Table S2.** Predicted estimates of child growth in Z-scores for interaction between religion and social group identities (linear specifications of outcomes). **Figure S1.** Proportion of sampled social groups in Hindus and Muslims in each NFHS wave. **Figure S2.** Heterogeneities in predicted prevalence of anthropometric outcomes by religion and social group interactions: 3 way interactions with other covariates. **Table S3.** Interaction between religion and caste on odds of anthropometric failures Stunting.

## Data Availability

All data and materials are publicly available.
