# Supplementary material for: When social identities intersect: understanding inequities in growth outcomes by religion- caste and religion-tribe as intersecting strata of social hierarchy for Muslim and Hindu children in India
Source: Int J Equity Health. 2023 Jun 14;22:115. doi: 10.1186/s12939-023-01917-3 (PMC10268355; doi:10.1186/s12939-023-01917-3)
Supplement: Supplementary file 1 — Additional file 1:Table S1. List of caste and tribal social groups that have been estimated in their intersections with two religious identities (Hindus and Muslims) as social strata jointly informing intersectional child health disparities. Table S2. Predicted estimates of child growth in Z-scores for interaction between religion and social group identities (linear specifications of outcomes). Figure S1. Proportion of sampled social groups in Hindus and Muslims in each NFHS wave. Figure S2. Heterogeneities in predicted prevalence of anthropometric outcomes by religion and social group interactions: 3 way interactions with other covariates. Table S3. Interaction between religion and caste on odds of anthropometric failures Stunting. [file 12939_2023_1917_MOESM1_ESM.docx]

**Supplementary Tables and Figures**

**Table S1:** List of caste and tribal social groups that have been estimated in their intersections with two religious identities (Hindus and Muslims) as social strata jointly informing intersectional child health disparities

| **Schedule Castes (SCs):** A list or schedule of deprived castes under the Constitution of India in 1950, who are considered the most discriminated historically “untouchable” castes. This list was originally created by the colonial administration in 1938. In independent India to date, only Hindus, Buddhists and Sikhs may be included as SCs. Thus, this is an umbrella term for several disadvantaged castes.  **Other Backward Classes (OBCs):** A group of socioeconomically deprived communities who were above the former “untouchable” castes in the caste hierarchy but are still considered socioeconomically backward. They became officially recognized in 1990. Members of all religions can be classified as OBCs. This is a more flexible list than Schedule Castes and Schedule Tribes, since it has varied over time and between different states.  **Other castes:** The residual “other” castes, also described as “forward castes”, or “upper castes” in India. Here, “forward” and “upper” are not legally recognized terms. These terms are used in historical and contemporary scholarship on caste, popular parlance, and political discourse in India. In this dissertation, the terms “other” castes and “forward” castes are used alternatively.  **Schedule Tribes** **(STs):** A list or schedule of 744 indigenous tribes, recognized under the Constitution of India. This list was also originally created under colonially controlled India in 1938. Tribes from all religions can be included as STs. Thus, this is an umbrella term for several disadvantaged tribes. |
| --- |

**Table S2:**

(i)Predicted estimates of child growth in Z-scores for interaction between religion and social group identities (linear specifications of outcomes)

1. Unadjusted estimates: Without covariates


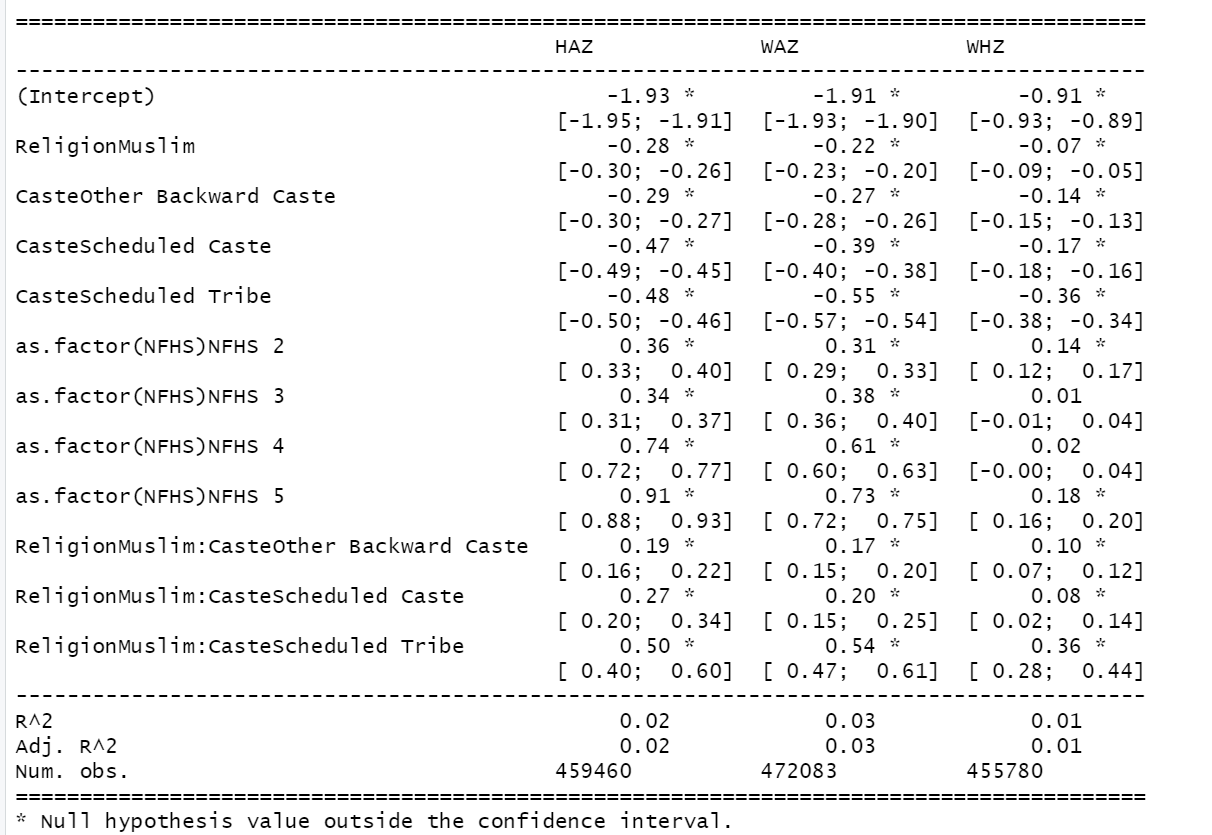


1. Adjusted estimates:These estimates are adjusted for household wealth, mother's education, mother’s height (for stunting and wasting), mother’s weight (for underweight and wasting), child’s age, child's sex, urbanicity. We also use state and district fixed effects, and included each NFHS survey wave as a fixed effect to control for all state invariant factors that may vary over time.


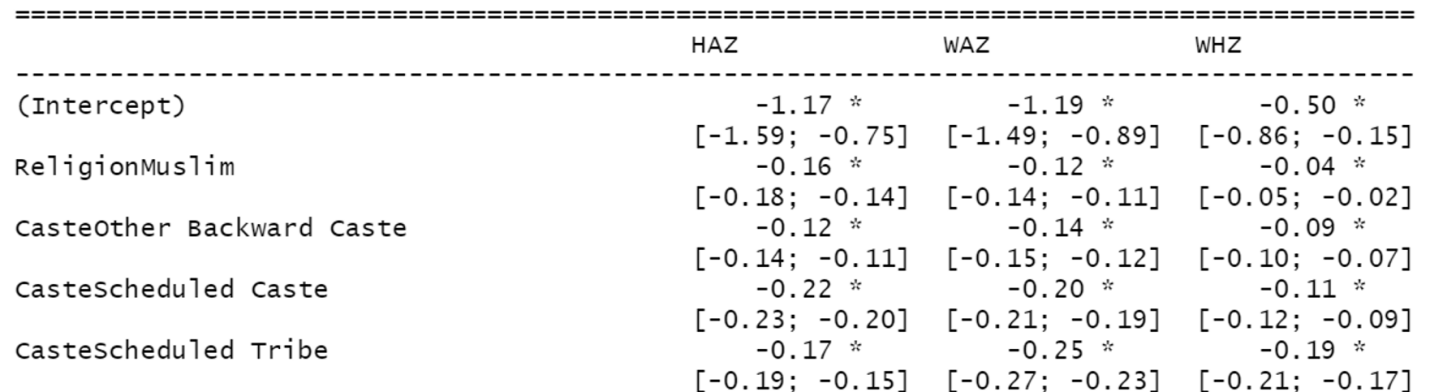


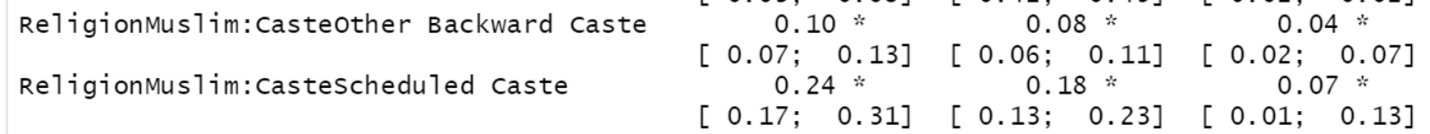


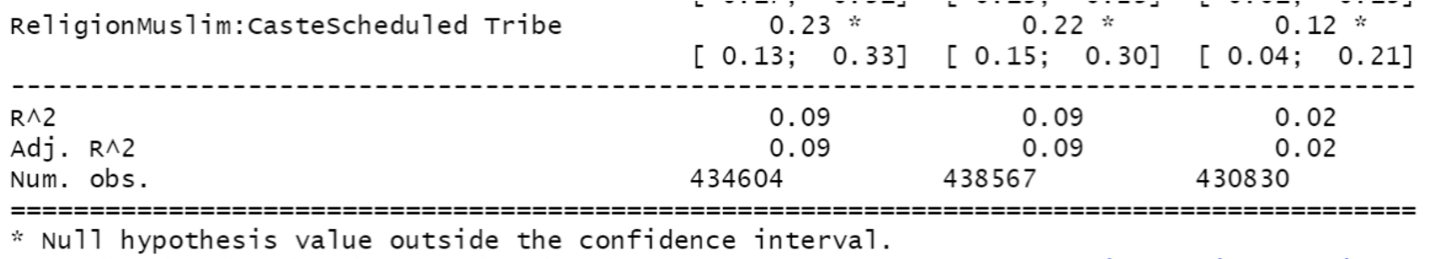


ii) Predicted estimates of anthropometric outcomes for interaction between religion and social group identities in OR scale (binary specifications of outcomes):

1. Unadjusted estimates :Without covariates


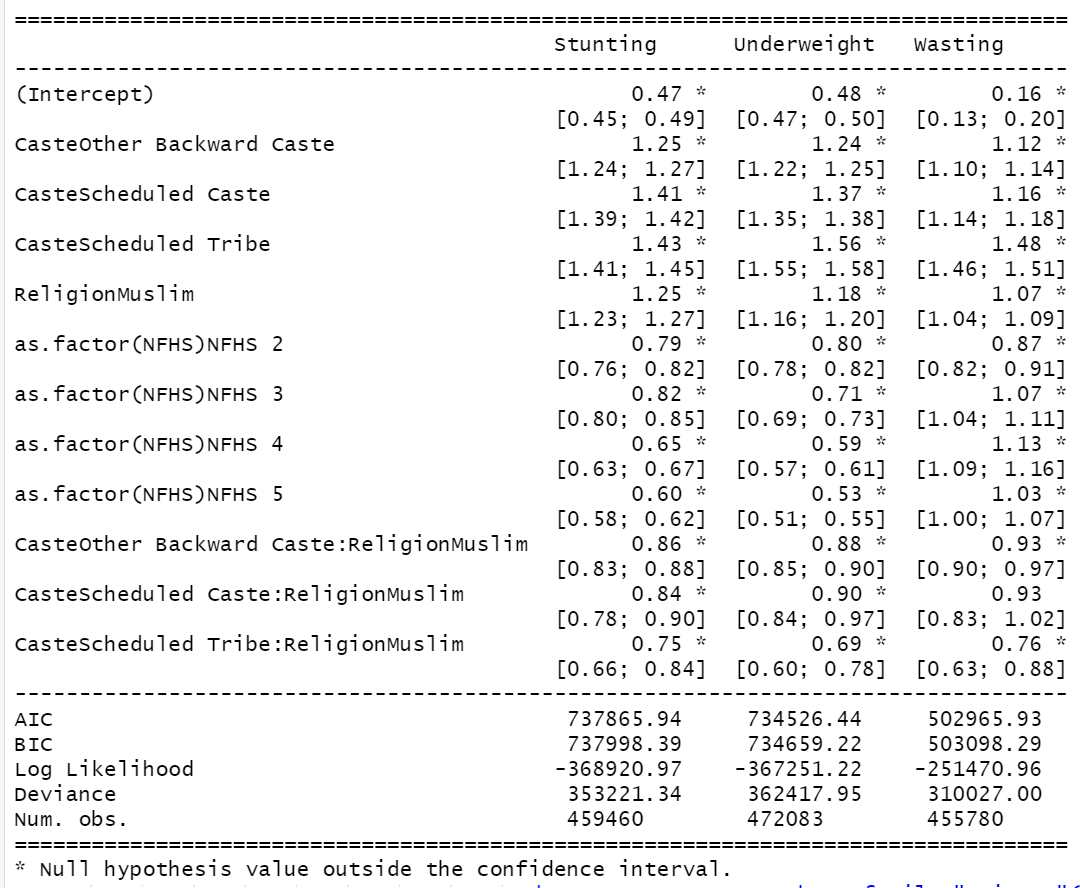


1. Adjusted estimates :These estimates are adjusted for household wealth, mother's education, mother’s height (for stunting and wasting), mother’s weight (for underweight and wasting), child’s age, child's sex, urbanicity. We also use state and district fixed effects, and included each NFHS survey wave as a fixed effect to control for all state invariant factors that may vary over time.


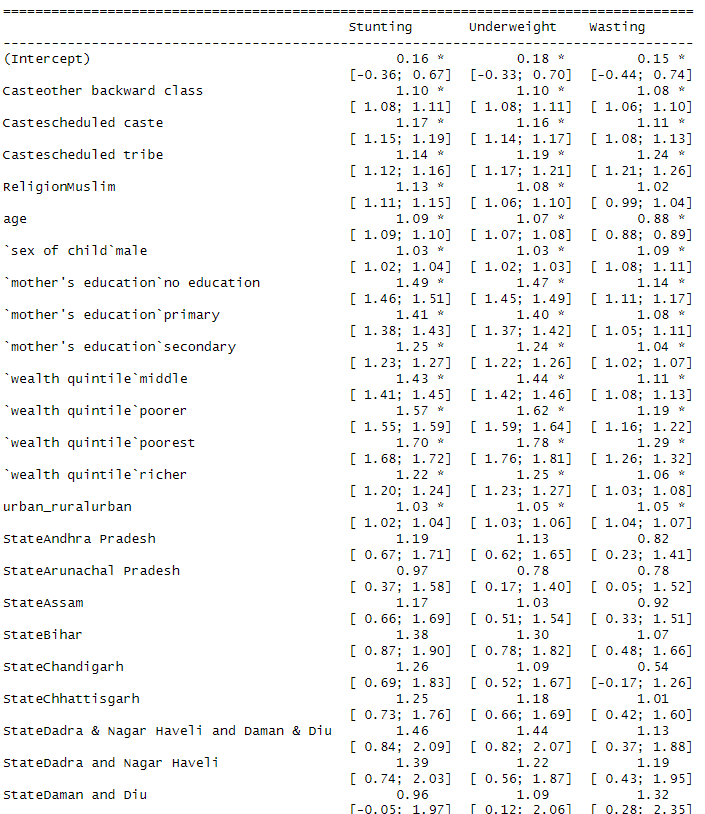


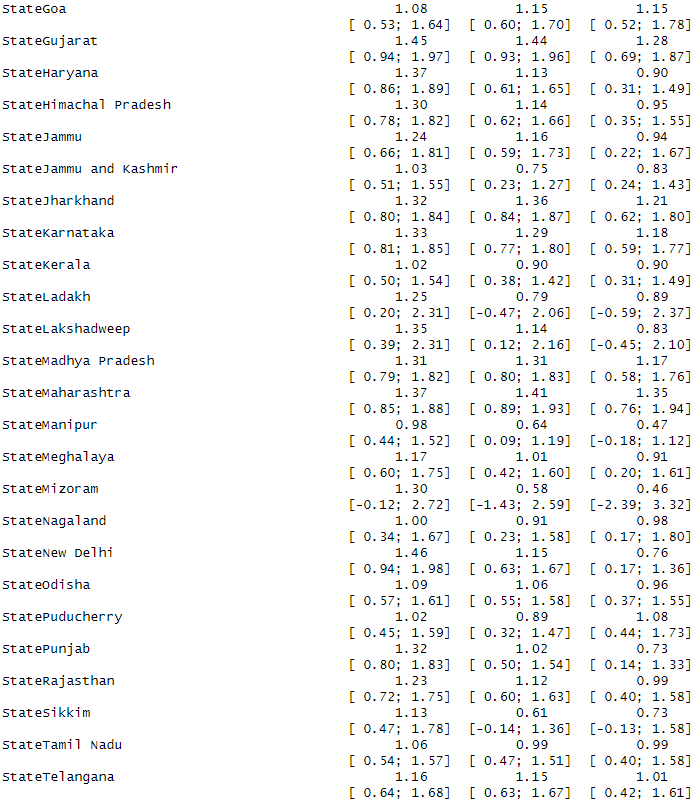


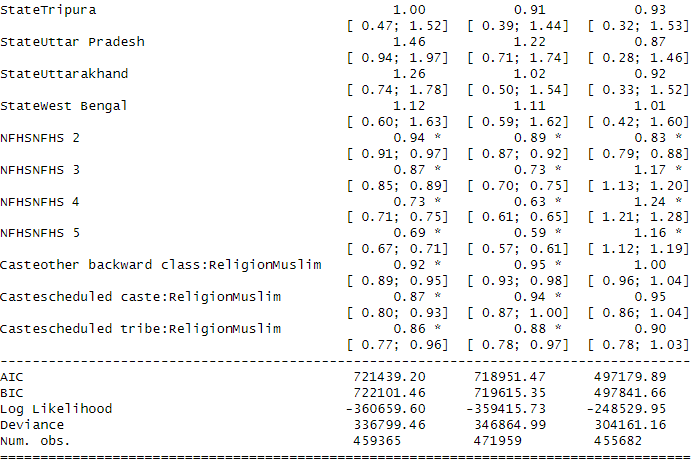


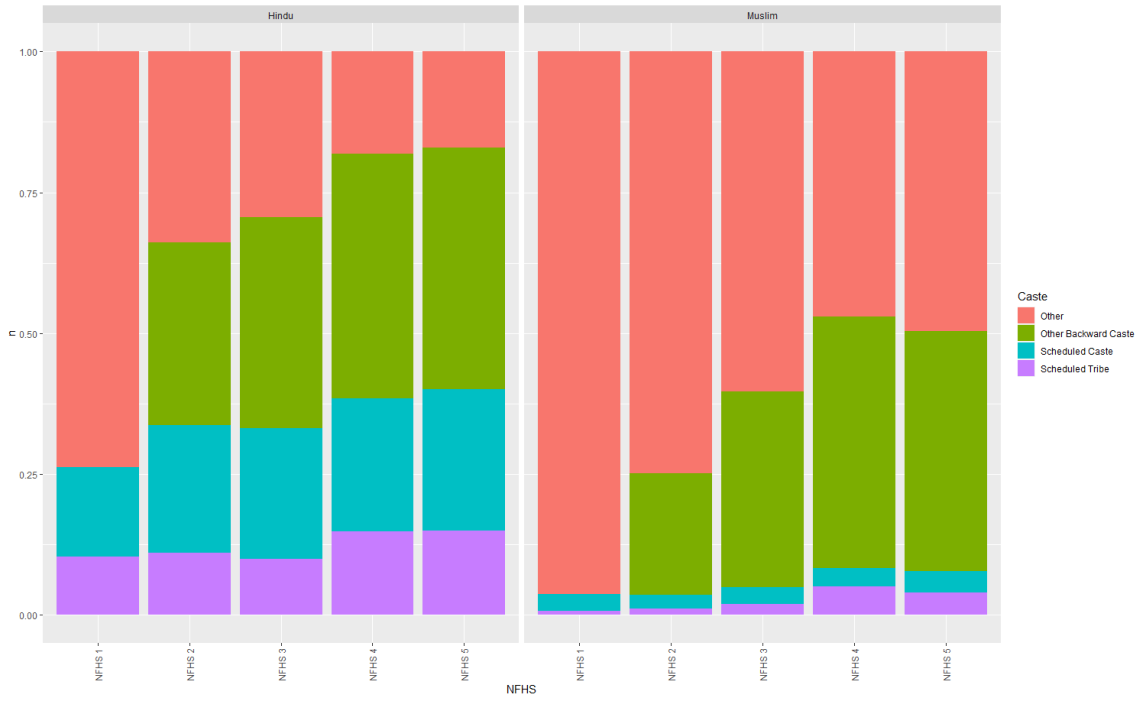


**Figure S1:** Proportion of sampled social groups in Hindus and Muslims in each NFHS wave

1. Gender of child


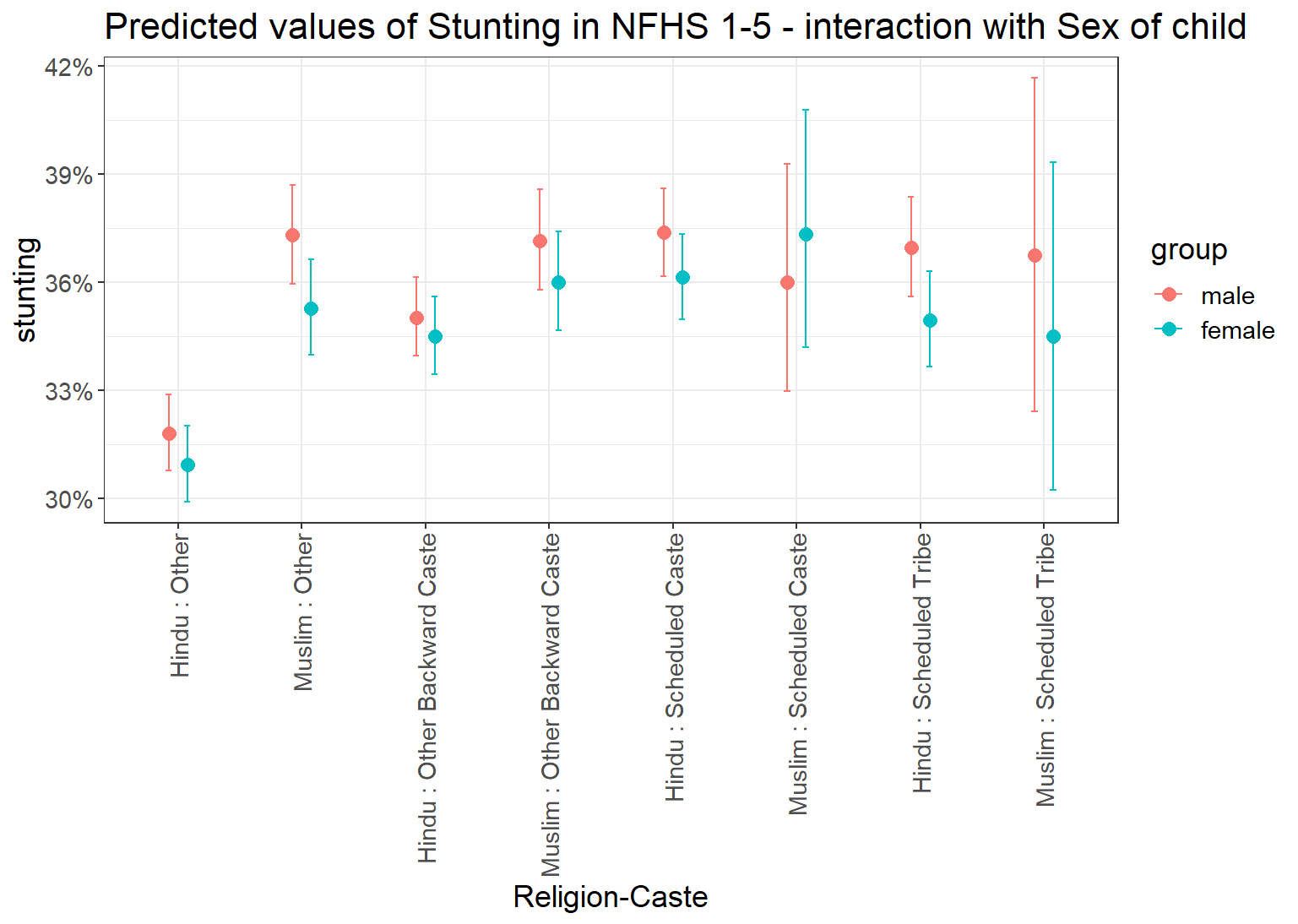


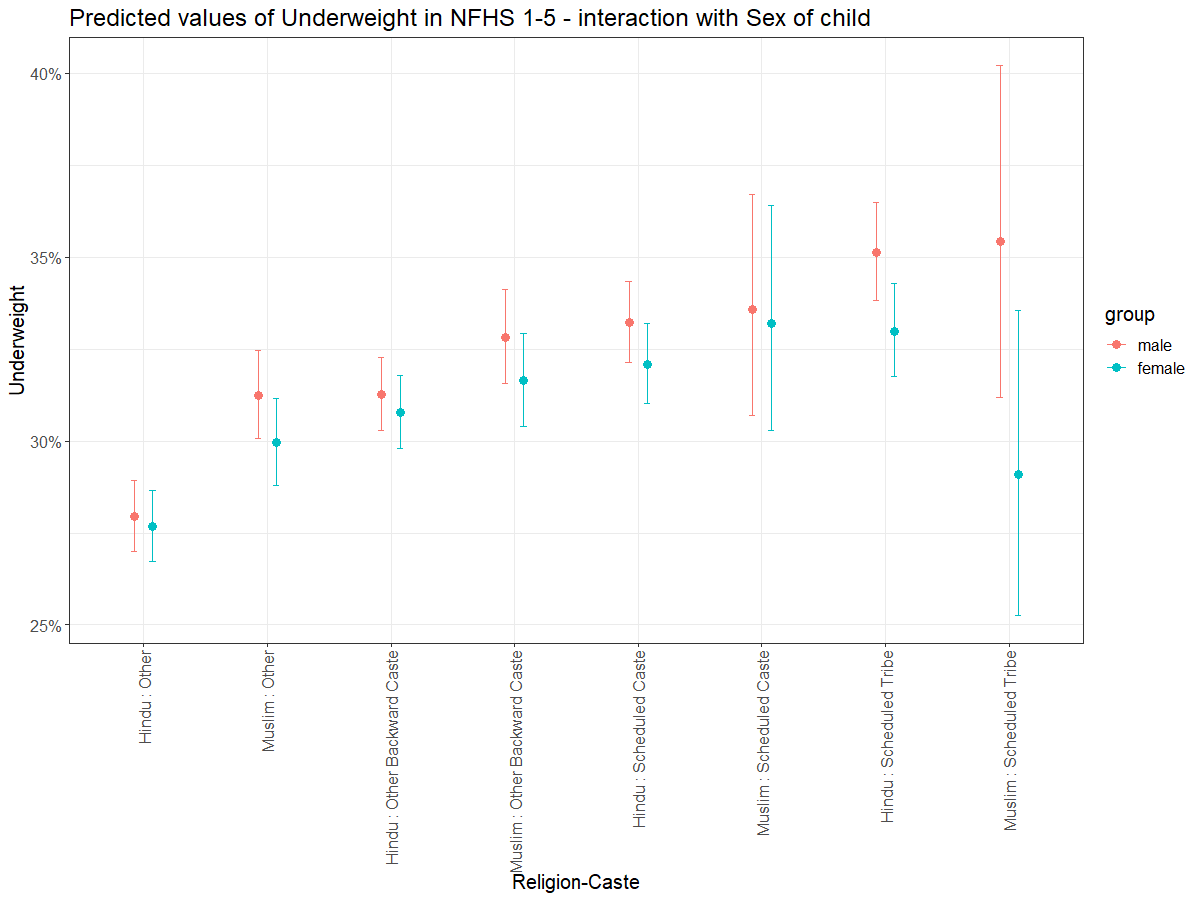


**
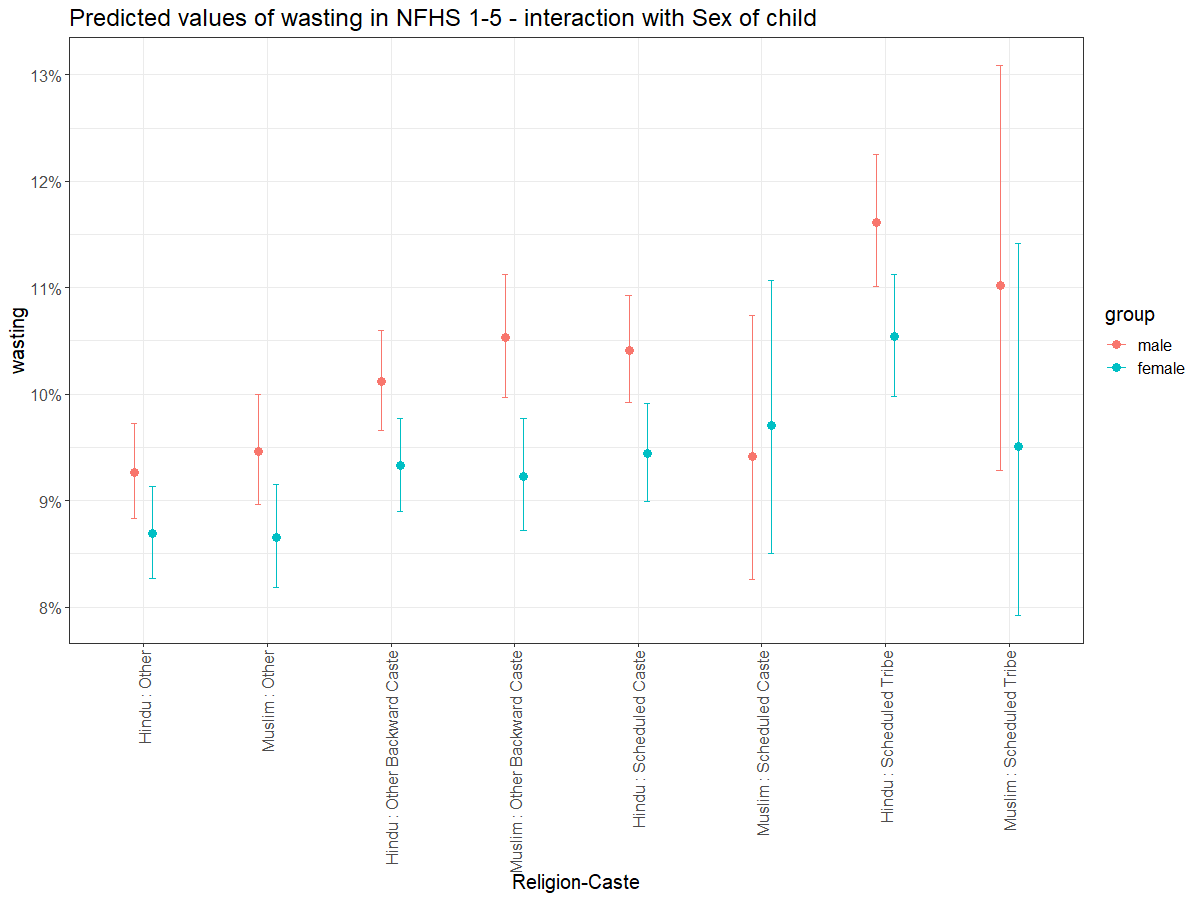
**

1. Age of child


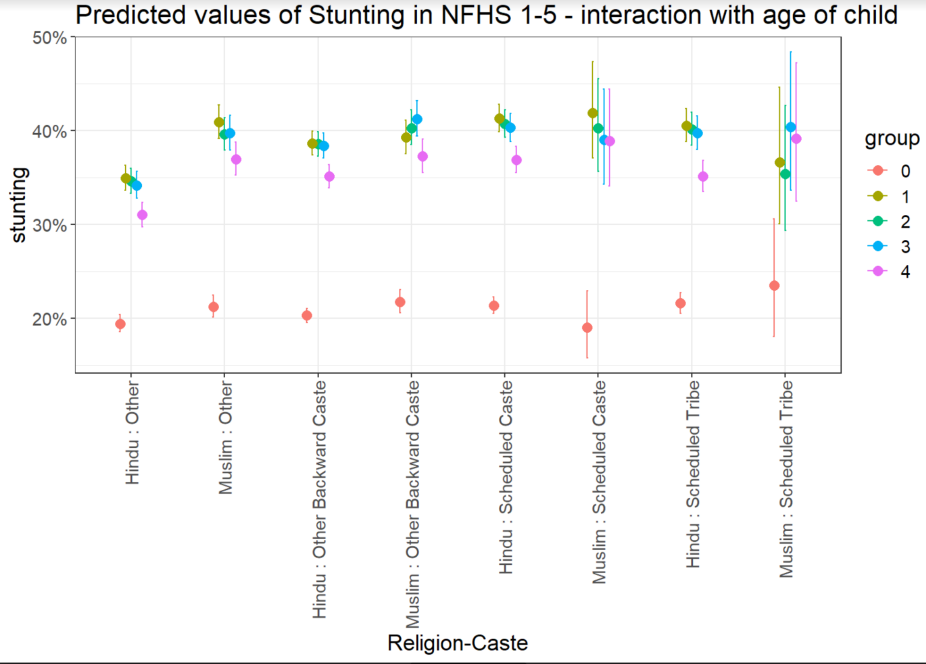


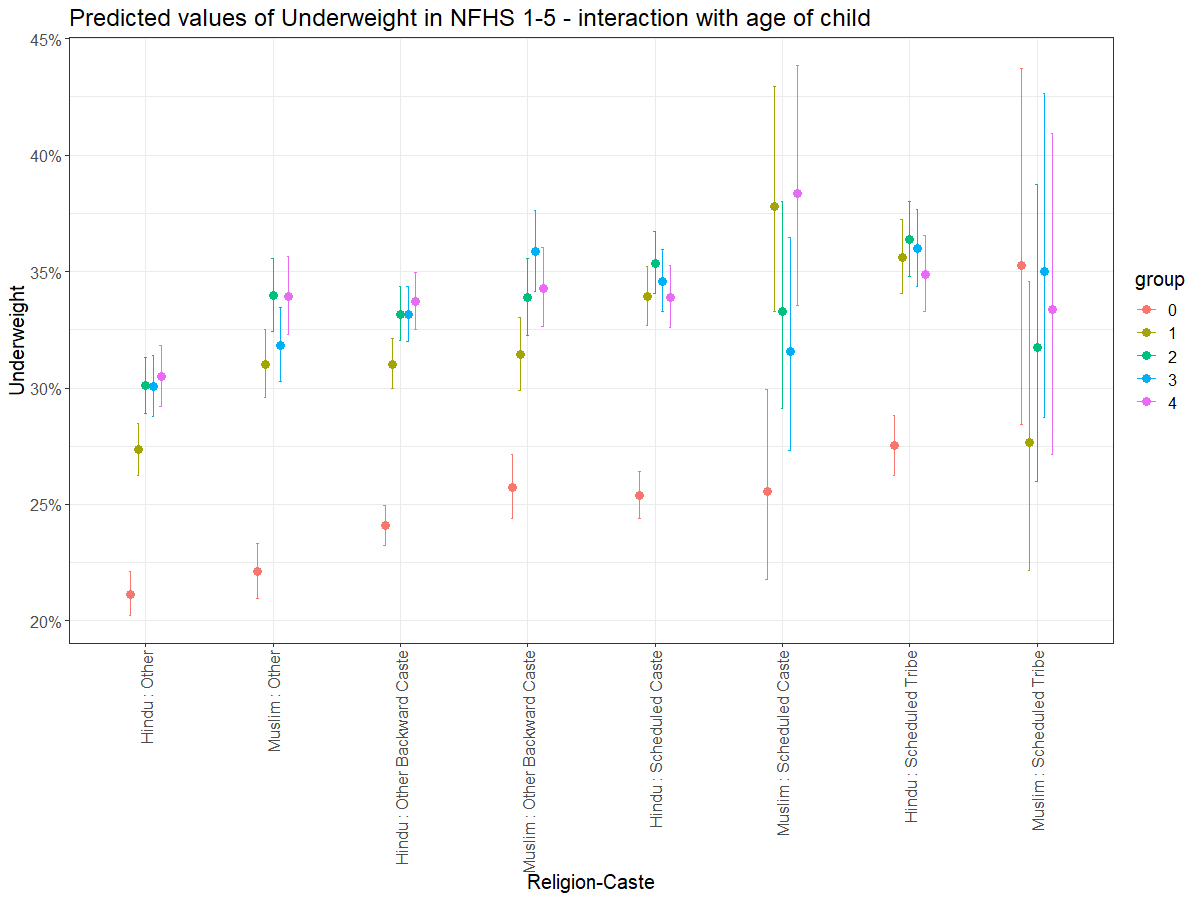


**
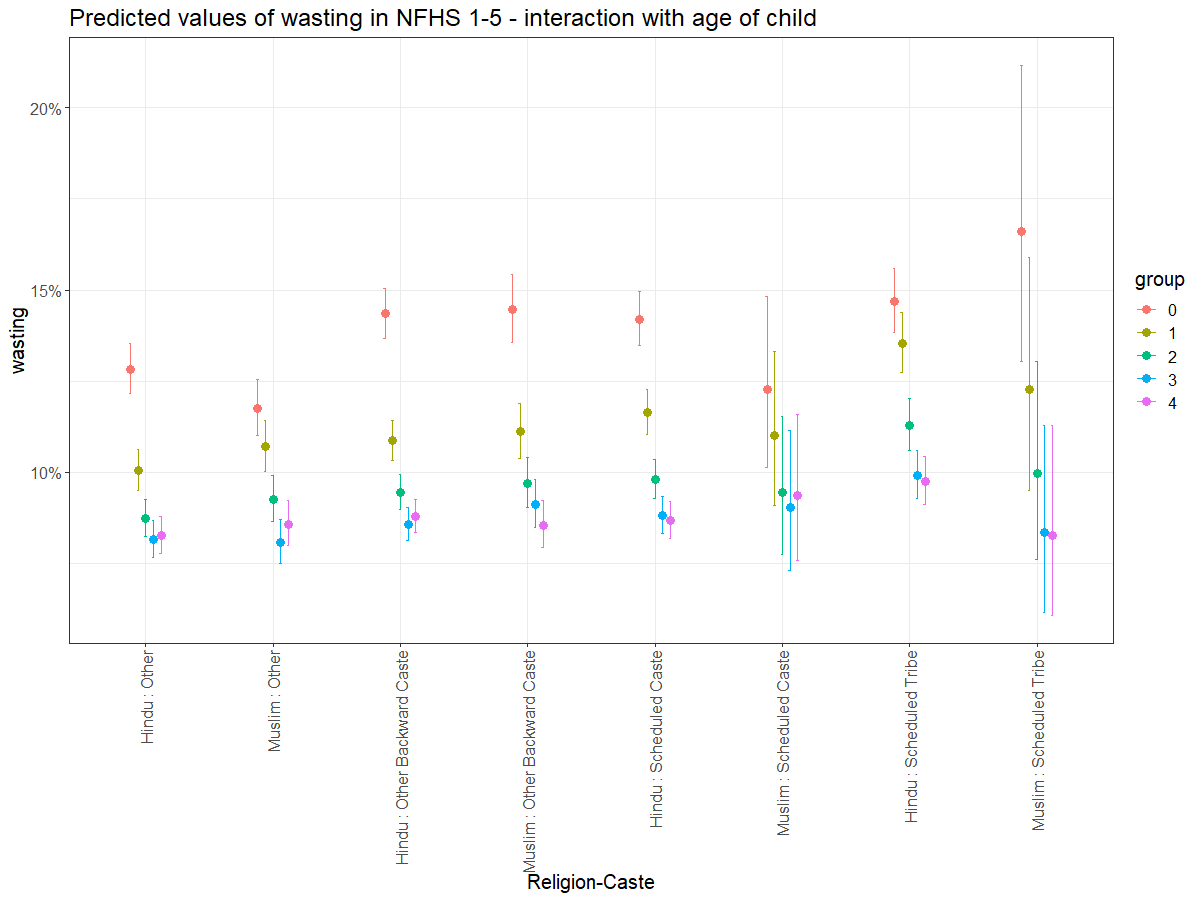
**

1. Household wealth quintile


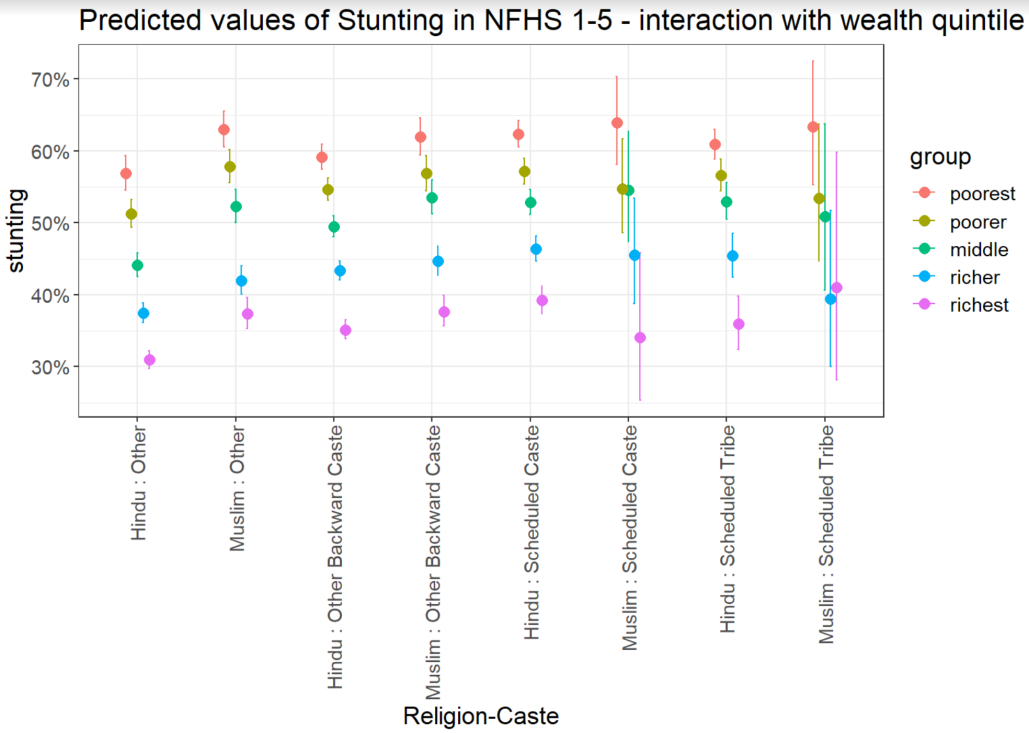


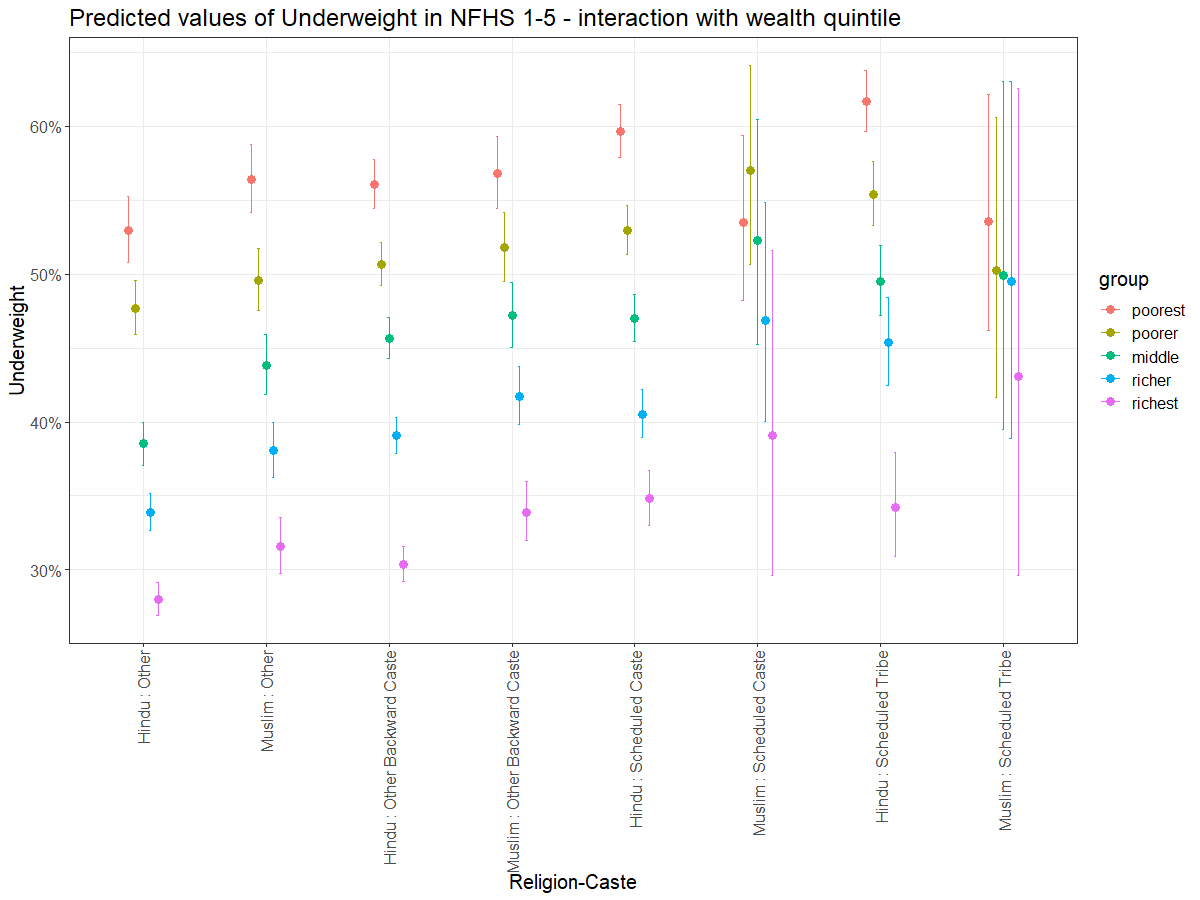


**
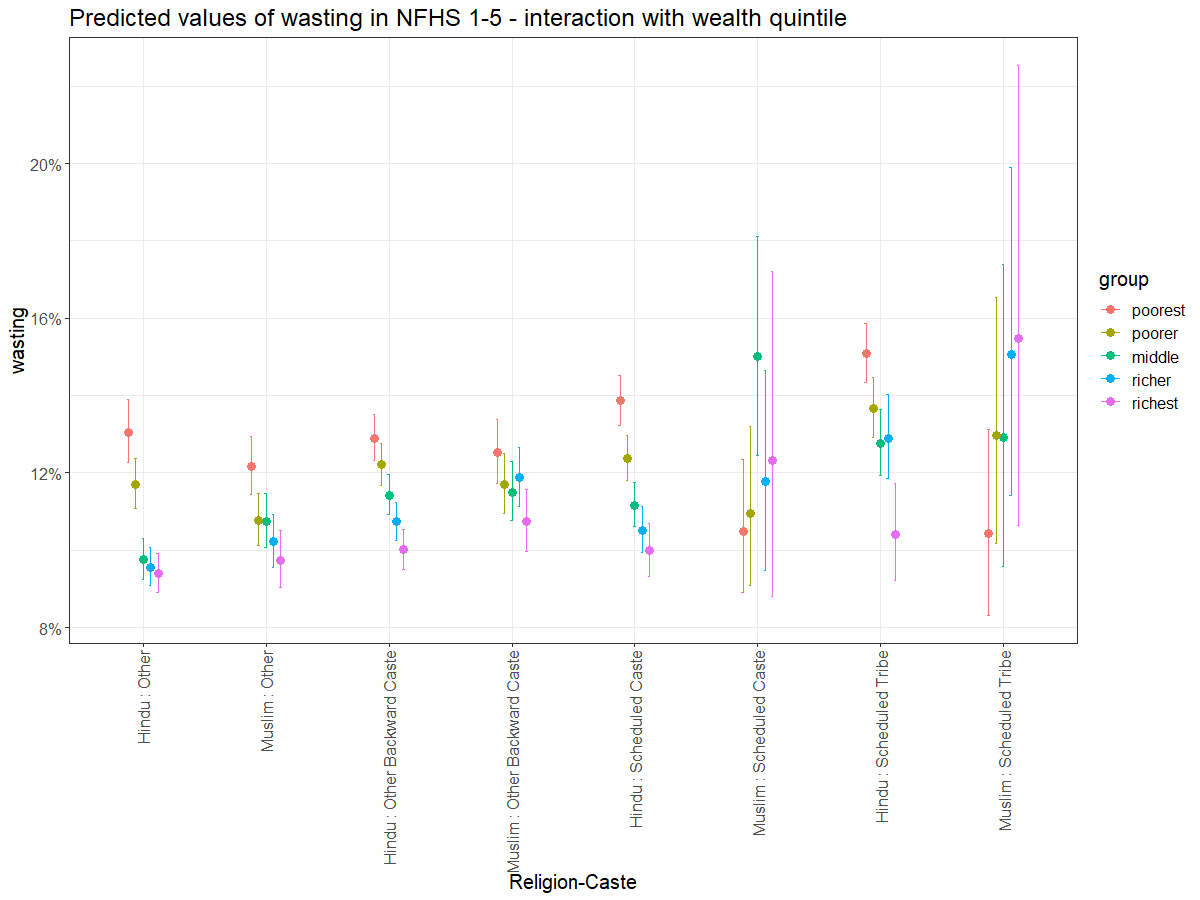
**

1. Mother’s education

**
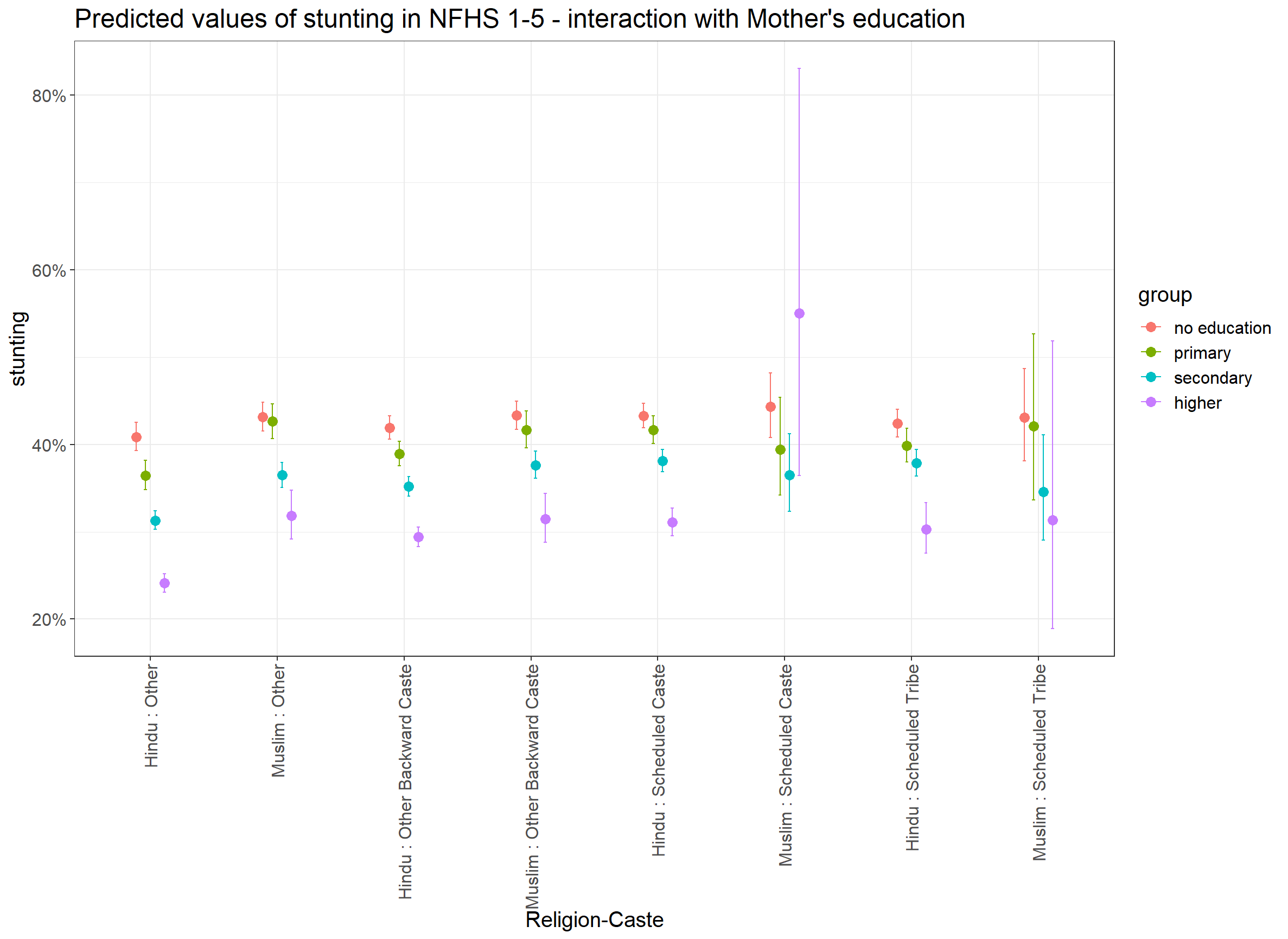
**


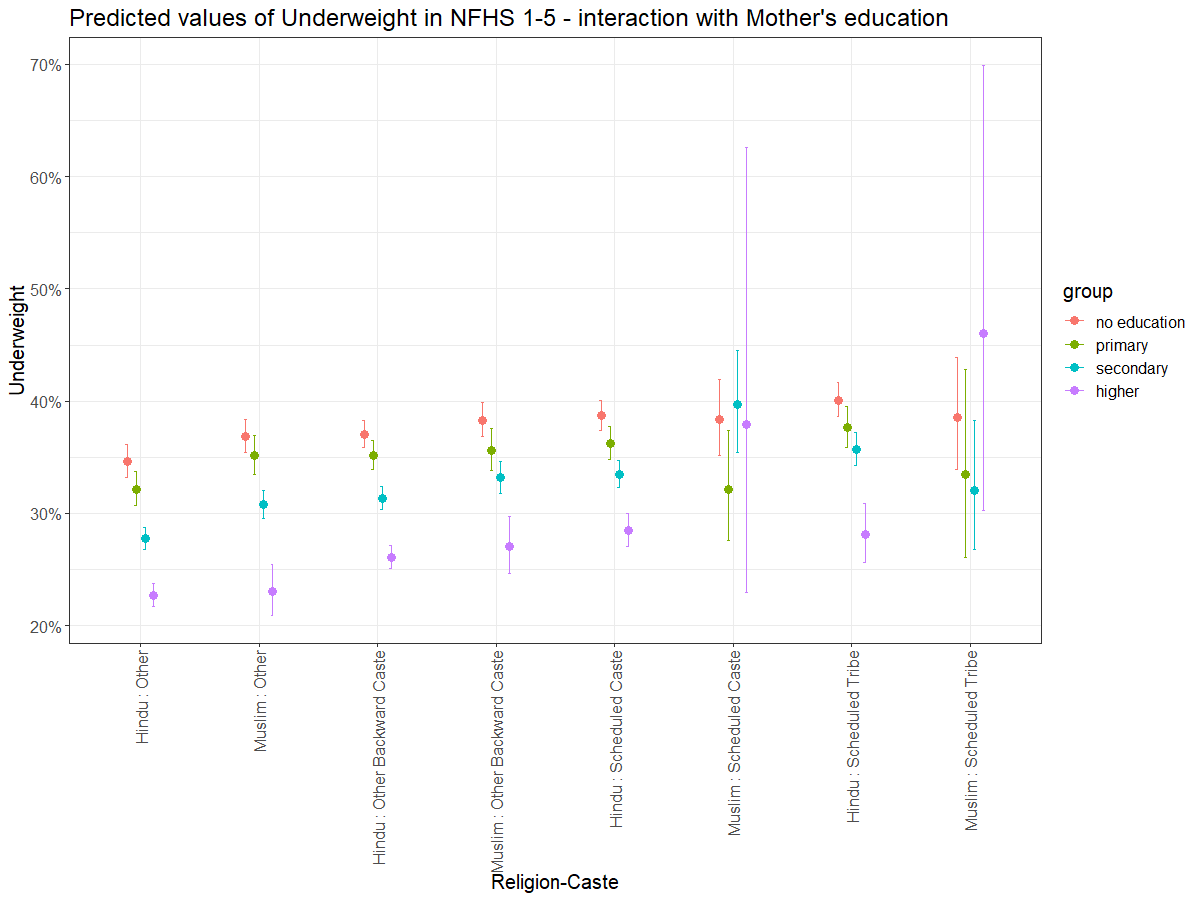


**
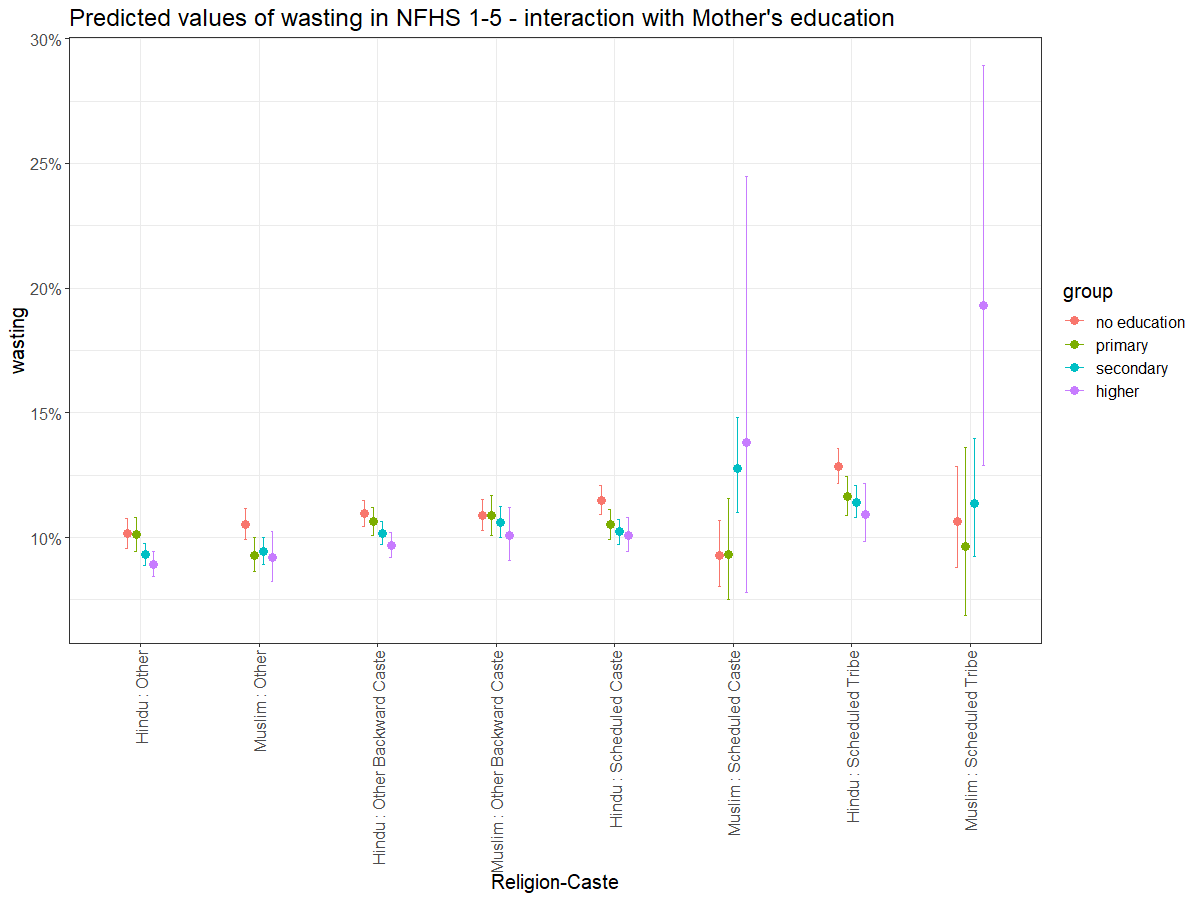
**

**Figure S2 :** Heterogeneities in predicted prevalence of anthropometric outcomes by religion and social group interactions: 3 way interactions with other covariates

**Table S3:** Interaction between religion and caste on odds of anthropometric failures Stunting

|  |  | **Caste** | | | | | | | | **RRs (95%CI) for Other Backward Class** | | **RRs (95%CI) for Scheduled Caste** | | **RRs (95%CI) for Scheduled Tribe** | |
| --- | --- | --- | --- | --- | --- | --- | --- | --- | --- | --- | --- | --- | --- | --- | --- |
|  |  | **Other Castes** | | **Other Backward Class** | | **Scheduled Caste** | | **Scheduled Tribe** | | **within strata** | | **within strata** | | **within strata** | |
|  |  | **N cases/total** | **RR (95%CI)** | **N cases/total** | **RR (95%CI)** | **N cases/total** | **RR (95%CI)** | **N cases/total** | **RR (95%CI)** | **of Religion** | **of Religion** | | **of Religion** | |  |
| Religion | Hindu | 20781/70281 | 1.0 | 58117/152516 | 1.11 [1.09, 1.13] p=1.07e-35 | 37106/86604 | 1.17 [1.15, 1.19] p=1.86e-73 | 21981/50844 | 1.15 [1.12, 1.17] p=1.525e-36 | 1.11 [1.09, 1.13] p=1.07e-35 | 1.17 [1.15, 1.19] p=1.86e-73 | | 1.15 [1.12, 1.17] p=1.52e-36 | |  |
|  | Muslim | 14068/37704 | 1.16 [1.13, 1.18] p=1.16e-40 | 12723/31131 | 1.17 [1.14, 1.19] p=1.15e-38 | 1077/2421 | 1.17 [1.1, 1.24] p=6.07e-07 | 1076/3103 | 1.14 [1.04, 1.24] p=0.01 | 1.01 [0.98, 1.03] p=0.54 | 1.01 [0.95, 1.07] p=0.78 | | 0.98 [0.9, 1.07] p=0.69 | |  |
| RRs (95%CI) for Religion within strata of Caste | |  | 1.16 [1.13, 1.18] p=1.16e-40 |  | 1.05 [1.03, 1.07] p=1.316e-07 |  | 1 [0.94, 1.06] p=0.92 |  | 0.99 [0.91, 1.08] p=0.85 |  |  | |  | |  |
| Measure of interaction on additive scale RERI: (95%CI) | | | | -0.1 [-0.13, -0.07] p=0.99 | | -0.16 [-0.23, -0.09] p=0.99 | | -0.17 [-0.27, -0.06] p=0.99 | |  | |  | |  | |
| Measure of interaction on multiplicative scale: Ratio of RRs (95%CI) | | | | 0.91 [0.88, 0.94] p=3.75e-11 | | 0.86 [0.81, 0.92] p=3.05e-06 | | 0.86 [0.78, 0.94] p=0.001 | |  | |  | |  | |
|  |  |  |  |  |  |  |  |  |  |  |  | |  | |  |

B) Underweight

|  |  | **Caste** | | | | | | | | **RRs (95%CI) for Other Backward Class** | **RRs (95%CI) for Scheduled Caste** | **RRs (95%CI) for Scheduled Tribe** |
| --- | --- | --- | --- | --- | --- | --- | --- | --- | --- | --- | --- | --- |
|  |  | **Other Castes** | | **Other Backward Class** | | **Scheduled Caste** | | **Scheduled Tribe** | | **within strata** | **within strata** | **within strata** |
|  |  | **N cases/total** | **RR (95%CI)** | **N cases/total** | **RR (95%CI)** | **N cases/total** | **RR (95%CI)** | **N cases/total** | **RR (95%CI)** | **of Religion** | **of Religion** | **of Religion** |
| Religion | Hindu | 11369/69829 | 1 [Reference] | 30164/151256 | 1.12 [1.1, 1.13] p=1.26e-37 | 17497/85957 | 1.17 [1.15, 1.2] p=2.06e-70 | 12838/50244 | 1.23 [1.2, 1.25] p=7.18e-78 | 1.12 [1.1, 1.13] p=1.26e-37 | 1.17 [1.15, 1.2] p=2.06e-70 | 1.23 [1.2, 1.25] p=7.185e-78 |
|  | Muslim | 6722/37304 | 1.1 [1.08, 1.13] p=6.75e-17 | 6066/30790 | 1.16 [1.13, 1.19] p=5.17e-33 | 471/2391 | 1.2 [1.13, 1.28] p=1.621e-08 | 559/3059 | 1.16 [1.06, 1.28] p=0.01 | 1.05 [1.03, 1.08] p=6.40e-05 | 1.09 [1.02, 1.16] p=0.01 | 1.06 [0.96, 1.16] p=0.25 |
| RRs (95%CI) for Religion within strata of Caste | |  | 1.1 [1.08, 1.13] p=6.75e-17 |  | 1.04 [1.02, 1.06] p=0.01 |  | 1.02 [0.96, 1.09] p=0.50 |  | 0.95 [0.86, 1.04] p=0.27 |  |  |  |
| Measure of interaction on additive scale RERI: (95%CI) | | | | -0.06 [-0.09, -0.02] p=0.99 | | -0.08 [-0.15, 0] p=0.97 | | -0.16 [-0.28, -0.05] p=0.99 | |  |  |  |
| Measure of interaction on multiplicative scale: Ratio of RRs (95%CI) | | | | 0.94 [0.92, 0.97] p=0.01 | | 0.93 [0.87, 0.99] p=0.02 | | 0.86 [0.78, 0.95] p=0.01 | |  |  |  |
|  |  |  |  |  |  |  |  |  |  |  |  |  |

1. Wasting

|  |  | **Caste** | | | | | | | | **RRs (95%CI) for Other Backward Class** | **RRs (95%CI) for Scheduled Caste** | **RRs (95%CI) for Scheduled Tribe** |
| --- | --- | --- | --- | --- | --- | --- | --- | --- | --- | --- | --- | --- |
|  |  | **Other Castes** | | **Other Backward Class** | | **Scheduled Caste** | | **Scheduled Tribe** | | **within strata** | **within strata** | **within strata** |
|  |  | **N cases/total** | **RR (95%CI)** | **N cases/total** | **RR (95%CI)** | **N cases/total** | **RR (95%CI)** | **N cases/total** | **RR (95%CI)** | **of Religion** | **of Religion** | **of Religion** |
| Religion | Hindu | 11369/69829 | 1 [Reference] | 30164/151256 | 1.08 [1.06, 1.11] p=3.91e-13 | 17497/85957 | 1.11 [1.08, 1.13] p=3.14e-17 | 12838/50244 | 1.23 [1.2, 1.27] p=2.03e-49 | 1.08 [1.06, 1.11] p=3.91e-13 | 1.11 [1.08, 1.13] p=3.14e-17 | 1.23 [1.2, 1.27] p=2.03e-49 |
|  | Muslim | 6722/37304 | 1.01 [0.98, 1.04] p=0.53 | 6066/30790 | 1.1 [1.07, 1.14] p=1.39e-09 | 471/2391 | 1.06 [0.97, 1.16] p=0.17 | 559/3059 | 1.14 [1.01, 1.29] p=0.03 | 1.09 [1.05, 1.13] p=8.99e-07 | 1.05 [0.96, 1.15] p=0.25 | 1.13 [1, 1.28]  p=0.04 |
| RRs (95%CI) for Religion within strata of Caste | |  | 1.01 [0.98, 1.04] p=0.53 |  | 1.02 [0.99, 1.04] p=0.21 |  | 0.96 [0.88, 1.05] p=0.39 |  | 0.93 [0.82, 1.05] p=0.23 |  |  |  |
| Measure of interaction on additive scale RERI: (95%CI) | | | | 0.01 [-0.03, 0.05] p=0.33 | | -0.05 [-0.15, 0.05] p=0.84 | | -0.1 [-0.25, 0.05] p=0.91 | |  |  |  |
| Measure of interaction on multiplicative scale: Ratio of RRs (95%CI) | | | | 1.01 [0.97, 1.05] p=0.70 | | 0.95 [0.87, 1.05] p=0.30 | | 0.92 [0.81, 1.04] p=0.18 | |  |  |  |
|  |  |  |  |  |  |  |  |  |  |  |  |  |
